# Metabolic Potential and Microbial Diversity of Late Archean to Early Proterozoic Ocean Analog Hot Springs of Japan

**DOI:** 10.1264/jsme2.ME24067

**Published:** 2025-07-23

**Authors:** Fatima Li-Hau, Mayuko Nakagawa, Takeshi Kakegawa, L.M. Ward, Yuichiro Ueno, Shawn Erin McGlynn

**Affiliations:** 1 Earth and Planetary Sciences Department, Institute of Science Tokyo, Tokyo, Japan; 2 Earth-Life Science Institute (ELSI), Institute of Science Tokyo, Tokyo, Japan; 3 Earth and Planetary Material Sciences, Tohoku University, Sendai, Japan; 4 Department of Geosciences, Smith College, Massachusetts, USA; 5 Blue Marble Space Institute of Science, Seattle, WA, USA; 6 Biofunctional Catalyst Research Team, RIKEN Center for Sustainable Resource Science, Wako, Japan

**Keywords:** microbial iron oxidation, Precambrian Earth, ferruginous environments, hot spring, early Earth primary productivity, metagenomics

## Abstract

Circumneutral iron-rich hot springs may represent analogues of Neoarchean to Paleoproterozoic oceans of early Earth, potentially providing windows into ancient microbial ecology. Here we sampled five Japanese hot springs to gain insights into functional processes and taxonomic diversity in these analog environments. Amplicon and metagenomic sequencing confirm a hypothesis where taxonomy is distinct between sites and linked to the geochemical setting. Metabolic functions shared among the springs include carbon fixation via the reductive pentose phosphate cycle, nitrogen fixation, and dissimilatory iron oxidation/reduction. Among the sites, Kowakubi was unique in that it was dominated by *Hydrogenophilaceae*, a group known for performing hydrogen oxidation, motivating a hypothesis that H_2_ as an electron donor may shape community composition even in the presence of abundant ferrous iron. Evidence for nitrogen cycling across the springs included N_2_ fixation, dissimilatory nitrate reduction to ammonia (DNRA), and denitrification. The low-salinity springs Furutobe and OHK lacked evidence for ammonium oxidation by ammonia monooxygenase, but evidence for complete nitrification existed at Kowakubi, Jinata, and Tsubakiyama. In most sites, the microaerophilic iron-oxidizing bacteria from the *Zetaproteobacteria* or *Gammaproteobacteria* classes had higher relative abundances than *Cyanobacteria*. Microaerophilic iron oxidizers may outcompete abiotic Fe oxidation, while being fueled by oxy-phototrophic *Cyanobacteria*. Our data provide a foundation for considering which factors may have controlled productivity and elemental cycling as Earth’s oceans became oxygenated at the onset of the Great Oxidation Event.

Putative evidence for life extends to some of the oldest graphitic inclusions, as far back as ~4.1 billion years ago (Gya), where carbon isotope ratios suggest metabolic activity (Bell *et al.*, 2015). However, little is known about the types of organisms that inhabited early Earth and the ecology expected (Crowe *et al.*, 2008; Planavsky *et al.*, 2011; Poulton and Canfield, 2011). One major control parameter differentiating contemporary and early Earth environments is atmospheric and oceanic oxygen concentrations. Multiple lines of evidence suggest that O_2_ was on the order of 1,000,000 times lower than present levels until the great oxygenation event (GOE) at around 2.4 Gya (Holland, 1986; Lyons *et al.*, 2014; Catling and Zahnle, 2020), which marks the transition from the Archean Eon to the Proterozoic Eon. This difference in oxygen concentration and the reasons behind it are critical for our understanding of biosphere productivity and early Earth’s ecosystems. In the low oxygen conditions of early Earth, the size of the biosphere would have been muted, perhaps 1000 times less than present day values (Ward *et al.*, 2019c). Biological O_2_ is generated through oxygenic photosynthesis, which today provides the largest flux of organic carbon and biomass at the Earth’s surface (Quéré *et al.*, 2018; Ward and Shih, 2019). Meanwhile as a substrate, oxygen serves as a powerful electron acceptor, allowing life to utilize a diverse range of electron donors as power sources (Amils, 2011). The accumulation of oxygen in the atmosphere at around 2.4‍ ‍Gya provoked changes on Earth’s mineralogy and metal reservoirs that opened new metabolic possibilities and may have been a springboard leading to the contemporary biosphere (Raymond and Segrè, 2006), making the transitory period of the GOE of great interest. This brings forward the question of how early microbial communities utilized the increasing concentrations of oxygen alongside other electron donors and how aerobes and microaerophiles interacted with anaerobic community members.

Here we focus on Japanese ferrous (Fe^2+^) iron carbonate rich hot springs to gain perspective on the types of communities and ecosystem processes which may have been operative as the Earth became oxygenated. In a similar way that terrestrial analogs are valuable for theorizing about other planetary bodies, analogue environments are also valuable for considering early Earth environments which were very different from today. At these sites, one or more conditions similar to those on early Earth or other planets can be found (Léveillé, 2010). Ferrous iron-rich surface environments are scarce on modern Earth as present oxidizing conditions quickly convert it to its insoluble ferric (Fe^3+^) form. Because of this scarcity, our understanding of iron-rich environments expected on the early Earth is yet limited.

Some consensus exists on the geochemical constraints that shaped Precambrian oceans: During the Archean, an anoxic (Cloud, 1968; Holland, 2002), ferruginous (~100‍ ‍μM Fe^2+^) (Planavsky *et al.*, 2011), circumneutral ocean was present (Krissansen-Totton *et al.*, 2018). Then, copious amounts of oxygen started accumulating globally at the onset of the GOE marking the Proterozoic (Catling, 2011). It has also been suggested that local “oxygen oases” could have existed after the evolution of oxygenic photosynthesis, but before the GOE (Anbar *et al.*, 2007). In search for environments on today’s Earth which may recapitulate these conditions, stratified lakes with bottom iron-rich water layers have been studied to gain insights into ancient ecology and host microbial communities capable of exploiting iron as an electron donor/acceptor (Crowe *et al.*, 2011; Walter *et al.*, 2014; Camacho *et al.*, 2017). One limitation of these studies is that the conditions are achieved at depths in which phototrophy occurs only by specialized organisms capable of surviving on 1% of surface irradiance or less or with phototrophy being completely absent (Julià *et al.*, 1998; Lehours *et al.*, 2007; Romero-Viana *et al.*, 2010; Descy *et al.*, 2012). Amongst previous studies at surface environments (Pierson *et al.*, 1999; Inskeep *et al.*, 2005; Hegler *et al.*, 2012; Ward *et al.*, 2017; Clair *et al.*, 2019; Kotopoulou *et al.*, 2019; Ward *et al.*, 2019a), community composition and activity varies. For example, at the ferrous iron-rich Chocolate Pots spring (Yellowstone National Park, USA), rapid abiotic iron oxidation outcompetes iron oxidizers (Clair *et al.*, 2017); at other sites, such as Crystal Geyser (Colorado, USA) (Probst *et al.*, 2018), the potential for iron cycling has been suggested but other metabolic pathways, centered on the high quantities of nitrates and sulfates seem to be more active; whereas in subsurface marine iron-rich systems, aerobic and anaerobic iron oxidizers are directly involved in nitrogen cycling (McAllister *et al.*, 2021). Here, we surveyed five iron-rich hot springs of Japan which could serve as analogues for Neoarchean to Paleoproterozoic oceans on the basis that they contain ferrous iron in ranges near what is‍ ‍expected in the Archean (~40–120‍ ‍μM) (Catling and Zahnle, 2020), have circumneutral pH, and have lower oxygen concentrations than modern atmosphere-equilibrated water. These variables, extremely rare today, position these springs as analogous to conditions which may have been expected as the early Earth became oxygenated. By comparing surficial iron-rich hot springs, we sought to increase our understanding of the role of Fe^2+^ as a driver of microbial community composition, investigating the geochemistry, microbial taxonomy membership, and bulk metabolic potential.

## Materials and Methods

### Study sites

**Fig. 1.**
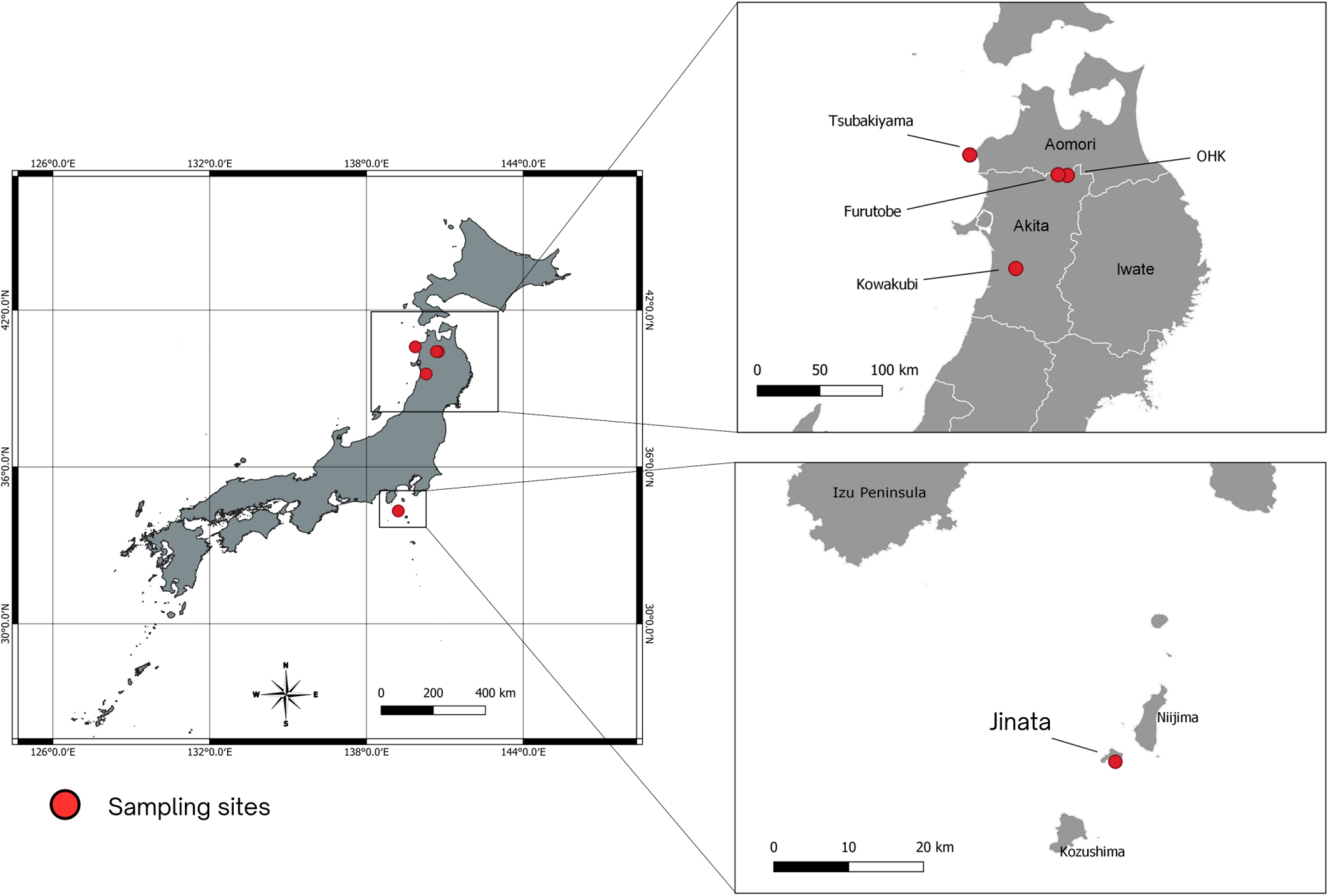
Map showing the location of hot springs in the study. Locations are marked with red circles.

**Fig. 2.**
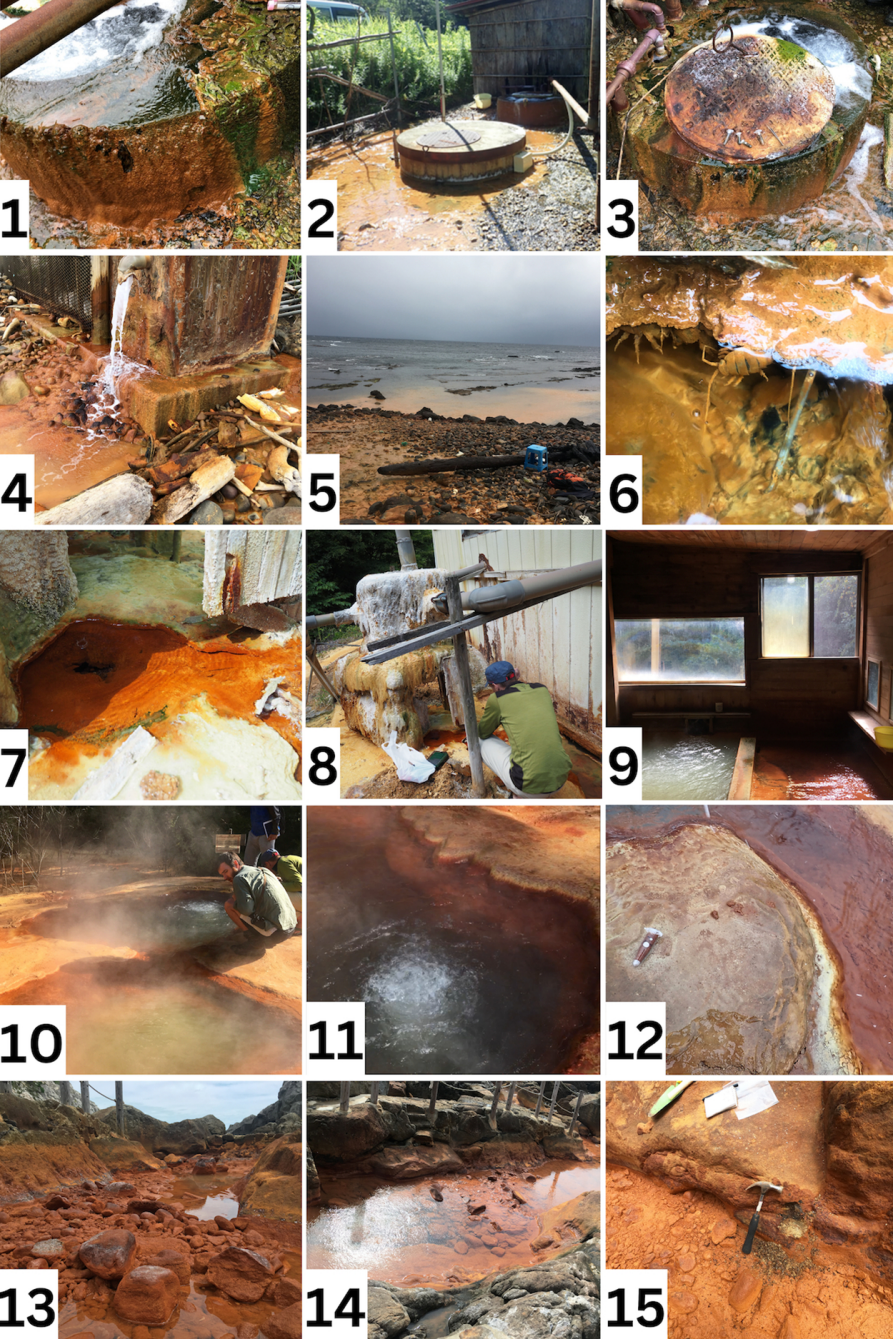
Pictures of the hot springs sampled in this study. Kowakubi hot spring [1, 2, and 3]: Source well showing microbial mats and iron oxides. Tsubakiyama hot spring [4, 5, and 6]: Spring water flowing from source tube, outflow of the spring to the ocean showing oxidation of fluid and close-up of source microbial mat. Furutobe hot spring [7, 8, and 9]: Spring water flowing from tube, pump to the bathing area, bathing area. OHK hot spring [10, 11, and 12]: Close-up to microbial mats and travertine deposition at the spring source. Jinata hot spring [13, 14, and 15]: Spring source during low tide, aerial view of spring at rising tide, sediments at low tide.

This study includes Jinata hot spring (34°19′04.4″N 139°12′57.3″E), Okuokuhachikuro (OHK) hot spring (40°24′28.66″N, 140°45′16.96″E), Tsubakiyama hot spring (40°35′39.72″N, 139°51′55.39″E), Furutobe hot spring (40°25′49.9″N 140°40′13.4″E), and Kowakubi hot spring (39°33′30.9″N 140°16′57.4″E). Jinata and Tsubakiyama are located on the coastline and flow directly to the North Pacific Ocean and the Sea of Japan, respectively. OHK, Kowakubi, and Furutobe hot springs are located inland (Fig. 1 and 2).

### Water geochemistry

In situ ana­lyses were conducted on source water. Dissolved oxygen (DO), salinity, conductivity, resistivity, total dissolved solids (TDS), and pH were measured with an Extech DO700 8-in-1 Portable Dissolved Oxygen Meter (FLIR Commercial Systems). For sulfide measurements, 0.5‍ ‍ml of water sample were reacted with 0.5‍ ‍ml of 30‍ ‍mM Zinc-Acetate solution in situ and kept at room temperature. Back in the laboratory, samples were analyzed with the Cline Reaction Assay Protocol (Cline, 1969) with variations to read on a multiplate reader (PerkinElmer) with translucent 96-well plates (Biolamo, AsOne). Ferrous iron was measured using the ferrozine assay (Stookey, 1970) with water samples reacted directly at the field site in 1.5‍ ‍mL tubes Eppendorf to avoid loss of Fe^2+^. Briefly, 600‍ ‍μL of source fluid were reacted with 160‍ ‍μL of ferrozine (1.2‍ ‍mM), 200‍ ‍μL of acetate buffer, and 40‍ ‍μL of ddH_2_O to a final volume of 1‍ ‍mL and kept away from sunlight. For laboratory ana­lyses, water samples were taken from the source avoiding aeration as much as possible. Water for geochemical ana­lysis, including for isotope values of dissolved inorganic carbon (DIC) and dissolved organic carbon (DOC), was collected using 50-mL syringes, passed through a 0.22‍ ‍μm pore size filter (Grade GF/F, Whatman), and kept on ice in evacuated, baked 50‍ ‍mL crimped glass vials for 24–48‍ ‍h until arrival to the laboratory and stored in a cold room at 5°C afterwards. For DIC/DOC concentration and stable carbon isotope ana­lysis, water was subsampled (5‍ ‍mL) replacing the removed volume with nitrogen gas to prevent sample oxygenation into 10-mL pre-evacuated and baked glass vials. Headspace CO_2_ values for DIC were measured after acidification with a few drops of H_3_PO_4_. After measurement, carbonate-free water was reacted with 0.1‍ ‍g sodium persulfate for DOC ana­lysis. The DOC concentration of acidified water samples were determined using a TOC analyzer (TOC-5000; Shimadzu, Kyoto, Japan). CO_2_ values for DIC concentration and *δ* ^13^C values of DIC and DOC were measured using an Agilent 6890 N gas chromatograph attached to a Thermo-Finnigan Delta XPPlus. *δ *^13^C Viena Pee Dee Belemnite (VPDB) values were calibrated against standardized samples ranging from 20.6 to 2.05‰ including international standard NBS19. The standard deviations were determined by 3 or more measurements.

### Amplicon microbial community ana­lysis

**Fig. 3.**
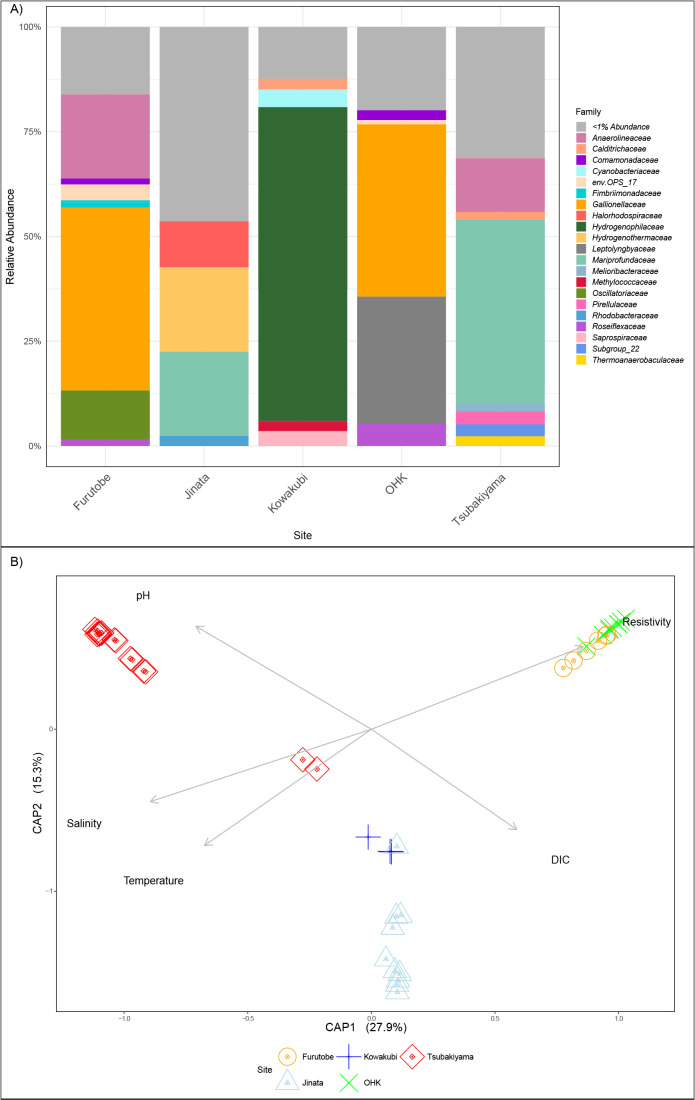
(A) Abundance of Families recovered from Illumina MiSeq 16S rRNA sequencing with primers 515FB and 926R from sediment and pore water at the source for OHK, Furutobe, Kowakubi, and Tsubakiyama. For Jinata hot spring, sediment and filtered water from a full tidal cycle and Ward *et al.* (2017) sediment sample sequences are included. (B) Canonical ana­lysis of principal coordinates (CAP) of Bray-Curtis distances and salinity, temperature, pH, DIC, and resistivity.

Depending on availability, ~800‍ ‍mg of sediment mixed with pore water or microbial mat samples mixed with spring water from Tsubakiyama, Kowakubi, Furutobe, and OHK hot springs were collected in triplicate and placed in ZR BashingBead^TM^ Lysis Tubes (Zymo Research). Details on each sample can be found in Table S1. Samples were amended with 750‍ ‍μL of Zymo Terralyzer BashingBead Matrix and DNA/RNA Shield (Zymo Research) to a final volume of ~2‍ ‍mL to preserve the DNA. Samples were processed for cell lysis for ~1‍ ‍min by attaching the tubes to a rechargeable reciprocating saw (Makita) with tape. The tubes were kept on ice until returning to the laboratory (24–48 hrs.). The samples were taken by either scrapping the microbial mat from the rock surface or scooping the sediment into the tubes with autoclaved spatulas, depending on the spring and mat conditions. The environmental DNA was extracted and purified after returning to the lab with a Zymo Soil/Fecal DNA extraction kit. Quantification of DNA was performed with a Qubit 3.0 fluorometer (Life Technologies) according to the manufacturer’s instructions. Purified DNA was submitted to the Integrated Microbiome Resource (Dalhousie University Halifax) for library preparation and sequencing following established protocols (Comeau *et al.*, 2011) with Illumina MiSeq i100. Extracted DNA was amplified using the 515FB (5′-GTGYCAGCMGCCGCGGTAA-3′) and 926R (5′-CCGYCAATTYMTTTRAGTTT-3′) primer pair targeting the V4–V5 regions of the 16S rRNA gene (Parada *et al.*, 2016; Walters *et al.*, 2016). For DNA extraction from water samples at Jinata, spring water (50‍ ‍mL) during low tide, rising tide, and high tide (over two tidal cycles) was sampled to account for variability in the water column. Source water was filtered using cellulose 0.22‍ ‍μm pore size filters (Sigma-Aldrich). The filters were kept on ice and DNA was extracted and sequenced using the aforementioned protocols. Sediment data from the Ward *et al.* (2019, accession numbers SRR7905026 and SRR7905030) study were pooled with water column data before a downstream ana­lysis. An initial 45 samples (Tsubakiyama, *n*=16; Kowakubi, *n*=3; Furutobe, *n*=6; OHK, *n*=6; Jinata, *n*=14) were analyzed. A sample list can be found in Table S1. Downstream ana­lysis was performed using the QIIME2 (ver. 2021.4) (Caporaso *et al.*, 2010) package, including the DADA2 (Callahan *et al.*, 2016) plug in for singleton removal and denoising. Sequences were clustered into operational taxonomic units (OTUs) with 99% similarity using an open-reference OTU picking protocol based on the Silva-138 database (Quast *et al.*, 2013). Taxonomic identification for each representative sequence was assigned using the Silva-138 database. 16S rRNA gene sequences were aligned using MAFFT (Katoh and Standley, 2013) and a phylogeny constructed using FastTree (Price *et al.*, 2009). OTUs identified as Unassigned (*n*=3) and Eukaryota (*n*=2) were removed. Unassigned sequences were identified using BLAST (Altschul and Lipman, 1990). Datasets were rarefied to 5,000 amplicon sequence variants (ASVs) to normalize the number of sequences per sample, samples with less than the normalization level were dropped from downstream ana­lysis. Dropped samples were JF12, JF19, JF34, JF41, and JF5 from the water column at Jinata during high tide events, one rising tide and one low tide event, likely due to low DNA concentration in the samples. Relative abundance figures were generated using the phyloseq (McMurdie and Holmes, 2013), qiime2R, vegan (Oksanen *et al.*, 2013), and ggplot 2 packages in R (Wickham and Wickham, 2007; Bisanz, 2018; Team, 2021) by pooling all samples from each site (Fig. 3A). Figures on the relative abundances per sample and site for Bacteria and Archaea can be found in Fig. S1A and S1B.

To compare beta diversity, Bray-Curtis distances and weighted/unweighted UniFrac distances were calculated and analyzed using Principal Coordinate Analysis (PCoA) (Fig. S1C, S1D, and S1E). The unweighted UniFrac distance considers only species presence/absence and measures the fraction of branch length unique to either community, while the weighted UniFrac distance incorporates species abundance, weighting the branch length based on abundance differences (Chen *et al.*, 2012). In order to understand the role of geochemistry in the clusters observed in beta diversity, we performed a canonical ana­lysis of principal coordinates (CAP) of Bray-Curtis distances (Fig. 3B).

### Metagenome functional ana­lysis

DNA extracted from the samples mentioned above was submitted to the Integrated Microbiome Resource (Dalhousie University Halifax) for metagenome sequencing with 2×150 bp Illumina NextSeq, with the exception of Jinata hot spring, for which only‍ ‍Ward *et al.* (2019a) sediment data (Accession number: SRR7905022) for the source pool was used. Raw sequence reads were quality controlled and trimmed of adapters using KneadData from the bioBakery suite (McIver *et al.*, 2018). All files were concatenated by site and assembled using MegaHit v 1.1.3 (Li *et al.*, 2015) within the MetaWRAP pipeline (Uritskiy *et al.*, 2018). Assembled contigs were filtered to a minimum length of 2,000 bp. For overall functional ana­lysis, filtered sequences were analyzed with the DiTing pipeline (Xue *et al.*, 2021). Briefly, contigs were annotated using prodigal (Hyatt *et al.*, 2010), and protein sequences were searched using Hmmsearch (Eddy, 2011) against KofamKOALA and DMSP databases (Aramaki *et al.*, 2020; Xue *et al.*, 2021). BBMap (Bushnell *et al.*, 2017) was used for relative abundance calculations. Functional ana­lysis on the iron cycling was performed using FeGenie (Garber *et al.*, 2020). In this study, gene distribution was determined based on scores obtained from a Canonical Correspondence Analysis (CCA), which was performed to examine the relationships between gene abundances and geochemical variables. The CCA scores were used to assess how well each gene correlated with the geochemical variables across different sites.

For metagenome-assembled genomes (MAGs), filtered contigs were binned using the MetaWRAP Binning module with Maxbin2 v 2.2.6 (Wu *et al.*, 2016) and Metabat2 v 2.12.1 (Kang *et al.*, 2019). In the BinRefinement module of MetaWRAP, bins were consolidated using the files generated from both assemblers. Quality and contamination were checked using CheckM within KBase (Parks *et al.*, 2015; Arkin *et al.*, 2018). Taxonomy was assigned using GTDB-Tk (Chaumeil *et al.*, 2022). MAGs were selected for further ana­lysis if contamination was ≤5% and completeness ≥60%. Further functional ana­lysis was performed using KBase with DRAM v 0.1.2 annotation (Shaffer *et al.*, 2020), MicroTrait BioElement v 1.9.1 pipeline ana­lysis (Karaoz), and RASTtk v‍ ‍1.073 annotation (Brettin *et al.*, 2015). Additional annotation was done with KEGG-KOALA, KEGG decoder, and FeGenie (Graham *et al.*, 2018; Aramaki *et al.*, 2020).

The sequenced data was deposited in the DNA Data Bank of Japan (DDBJ) under the accession number PRJDB18100, which can be accessed through the National Center for Biotechnology Information (NCBI) database.

## Results and Discussion

### Geochemistry at the iron-rich hot springs

All the springs in this study were micro-oxic, contained >40‍ ‍μM Fe^2+^, and were rich in DIC, positioning them as analogs to early Earth as it transitioned to an oxygenated environment (Table 1). For Jinata hot spring, geochemical values are represented as the mean of all values in a whole tidal cycle during high tide season to account for temporal variability in the water column. Averaged Fe^2+^ concentrations at Jinata and single-time measurements at OHK were lower than previously reported, with 42‍ ‍μM vs 261‍ ‍μM at Jinata and 72‍ ‍μM vs 114‍ ‍μM at OHK (Ward *et al.*, 2017, 2019a). Oxygen was also different compared to previous measurements: 75‍ ‍μM at Jinata here vs. 5‍ ‍μM previously, and 50‍ ‍μM at OHK here vs. less than 15‍ ‍μM previously. The average temperature of Jinata was lower as well: we recorded an average of 55°C, whereas previously a measurement of 63°C was recorded. Previously reported geochemical conditions at Jinata were given for only one time point. The highest temperature we recorded was 61.7°C, closer to the previously reported one. However, the spring is highly dynamic due to its tidal variation and connection with the ocean. The averaged measurements reported here reflect an overview of the conditions in the spring throughout a tidal cycle. Hot springs are dynamic environments (Meyer-Dombard and Malas, 2022), and continued observations will be of interest; differences in measurement methods (*e.g.* depth of measurement in the water column, time of day, weather conditions) or local changes can result in high variability in reported values. The coastal hot springs Jinata and Tsubakiyama presented higher salinity, suggesting a mixing of sea water with the hydrothermal fluid. Kowakubi hot spring also exhibited high salinity, likely from deep brine reservoirs. All springs showed enriched *δ*^13^C DIC, which has been observed in other springs where degassing of CO_2_-rich fluids occurs at the surface (Evans *et al.*, 2008). A hierarchical clustering ana­lysis (Fig. S1F) between the sites showed Jinata as a singleton cluster. Tsubakiyama and Kowakubi springs were grouped closely together. OHK and Furutobe are geographically close and geochemically grouped together.

### Microbial communities at the iron-rich hot springs

Amplicon sequencing was performed on sediment/pore water and microbial mats obtained as close to the source as possible to gain information on the microbial community structure at the springs. At Jinata hot spring, reads from the filtered water samples were pooled with the sediment samples to account for tidal variations in the spring. From the 45 samples, 4,466,250 total amplicon sequencing reads were generated. After quality control, 3,127,216 sequences were used for further ana­lysis. The OTU table is provided in Table S2. A total of 2,666 OTUs were defined at a 99% similarity cutoff using the Silva 138 database. Eukaryote (*n*=2) sequences and sequences unassigned at the Kingdom level (*n*=3) were removed. Two of the three unassigned sequences represented uncultured Archaea reported from the Mid-Okinawa Trough (Accession No. AB825905) (Yanagawa *et al.*, 2013) at Tsubakiyama and the Nankai Trough (Accession No. AB327833) (Hoaki *et al.*, 2019) at Jinata according to BLAST ana­lysis. After removal of unassigned sequences, 2,661 OTUs were obtained, from which 197 corresponded to Archaea and 2,464 to Bacteria. Because of the differences in library size, samples were rarefied (McMurdie and Holmes, 2014; Willis, 2019; Cameron *et al.*, 2020). After rarefaction, 2,154 OTUs were left, from which 165 corresponded to Archaea and 1,989 to Bacteria. Alpha diversity plots can be found in Fig. S1G. The observed richness was variable between the sites. Jinata and Tsubakiyama had higher observed richness, but also a higher sample number. Sample number has a strong and direct effect on the number of OTUs and interpretation about species richness between sites should be done with caution (Willis, 2019).

For Bacteria, all sites harbored unassigned OTUs at the Phylum level, suggesting yet to be understood diversity hidden in these hot springs. The total number of assigned bacterial:archaeal phyla were 52:12 at Jinata, 40:9 at Tsubakiyama, 23:2 at Kowakubi, 35:2 at Furutobe, and 25:2 at OHK. Although most universal primers underestimate Archaea (Bahram *et al.*, 2019), reamplification with Archaea-specific primers (A956F-A1401R) (Comeau *et al.*, 2011) showed no change in observed diversity (data not shown) and therefore only previously selected universal primers (515F-926R) (Walters *et al.*, 2016; Parada *et al.*, 2016) were used. Only Jinata and Tsubakiyama contained unassigned phyla from the domain Archaea (Fig. S1A and S1B). To compare beta diversity, Bray-Curtis and weighted/unweighted UniFrac distances were calculated and analyzed using PCoA, which showed high intra-site heterogeneity (Fig. S1C, S1D, and S1E). A canonical ana­lysis of principal coordinates of Bray-Curtis distances and geochemical parameters (Fig. 3B) showed that the distribution in the projected space of the biological clusters is driven strongly by salinity, temperature, DIC, and pH. Salinity and resistivity have an inverse relationship, with resistivity decreasing as salinity increases. Because both parameters describe the same environmental gradient, salinity was prioritized in the discussion to avoid redundancy. The ana­lysis revealed that salinity and temperature divide the samples in three clusters on Axis 1 (CAP 1). In Axis 2 (CAP 2), pH and DIC concentration influenced the distribution of high salinity samples. The beta diversity ana­lysis on the microbial communities in the sediment and microbial mats followed a similar pattern of distribution to that of the geochemistry, while the CAP ana­lysis confirmed salinity and temperature as dominant factors influencing distribution (Fig. 3B), followed by pH and DIC, according to the loadings obtained from the ana­lysis.

Except in Kowakubi, well-known groups of microaerophilic iron-oxidizing Bacteria were the most abundant organisms across the sites, either from the *Mariprofundaceae* family at Jinata (17.58%) and Tsubakiyama (47.3%) or the *Gallionellaceae* family at Furutobe (38.88%) and OHK (24.13%) (Fig. 3A). It is worth noting that the amplicon sequencing shows relative abundances and not absolute abundances of the groups (*i.e.*, not cell numbers). Despite this, our data suggests that the most abundant primary producers at these sites utilize iron as an electron donor. In the springs studied here, as in other ferrous iron-rich environments (Ward *et al.*, 2017, 2019a; Prondzinsky *et al.*, 2021) cyanobacteria are present in high abundances, further supporting that they can grow under such conditions. This contradicts previous studies that suggested iron was toxic to the group and that this could be a cause for the oxygenation delay before the GOE (Swanner *et al.*, 2015). It is unclear if the cyanobacteria or the iron oxidizers are the main primary producers as cell abundances do not necessarily relate to carbon fixation rate. Some studies have shown diurnal cycles can influence which autotrophic community members are most active (van‍ ‍der Meer *et al.*, 2007), whereas in marine snow, iron-oxidizing bacteria (FeOB) drive rates of carbon fixation with almost no carbon fixation from phototrophic community members (Li *et al.*, 2021). Experimental approaches to discern the primary production rates per taxonomic group are necessary, as they could have implications for the role of chemolithotrophs on early Earth’s productivity.

From the taxonomic abundances of the different groups found at the springs, it is obvious that there is no consistent microbial community assembly that can be attributed to iron-rich, microaerobic, circumneutral hot springs. Beta diversity ana­lyses revealed a separation between Jinata, Tsubakiyama and Kowakubi-type microbial communities and Furutobe, OHK-type microbial communities (Fig. 3B, S1C, S1D, and S1E). It has been previously reported that *Gallionellaceae* and *Mariprofundaceae* families have different salinity tolerances (McBeth *et al.*, 2013), although the role of temperature has not been discussed at length. In the case of these hot springs, salinity in Jinata, Kowakubi, and Tsubakiyama is one order of magnitude higher than in Furutobe and OHK at concentrations of ~2 to ~20 parts per thousand. Both Jinata and Tsubakiyama are located on the coast and it is likely that ocean water is involved in the rock-water interactions below the surface, providing not only a source for saline water, but also input for marine microbes, sulfate, and organic carbon. It is of note that the distance between these hot springs is more than 700‍ ‍km, and yet, the most abundant microbial groups present are microaerophilic iron oxidizers from the *Zetaproteobacteria*. However, they also harbor site-specific microbial groups:‍ ‍the *Gammaproteobacteria* (*Sulfurivirga sp.*) and *Aquificae* (*Persephonella sp.*) abundance at Jinata, and the higher abundance of *Chloroflexi* (*Anerolineaceae*) and *Alphaproteobacteria* from several families at Tsubakiyama point to different underlying metabolic and geochemical processes occurring. The second most abundant functional group found in Jinata are chemolithotrophs, utilizing either thiosulfate or hydrogen. At Tsubakiyama, the observed *Chloroflexi* and *Alphaproteobacteria* include members with‍ ‍a variety of metabolisms, including those capable of anoxygenic photosynthesis (Camacho *et al.*, 2017). At OHK, *Chloroflexi* from the aerobic heterotrophic *Herpetosiphonaceae* family and the *Chloroflexia* class have‍ ‍been reported (Ward *et al.*, 2021) and were also detected here. Furutobe and OHK are located in mountainous areas and in close proximity. The most abundant groups in these hot springs are the iron-oxidizing *Gallionellaceae* family and *Cyanobacteria*. However, Furutobe harbors *Cyanobacteria* of the *Oscillatoriaceae* family and high abundances of *Chloroflexi*, whereas OHK harbors mostly *Leptolyngbyaceae Cyanobacteria*. Kowakubi has a strikingly different microbial community composition from the other sites characterized in this study, suggesting the presence of mole­cular hydrogen in the hot spring. Kowakubi is dominated by *Hydrogenophilus sp.* and hydrogenotrophic methanogens, with 90.2% of the Archaea belonging to the Class Methanobacteria, though overall they represent only 0.12% of the whole microbial community.

### Functional profiles of microbial communities and species at the iron-rich hot springs

To study the metabolic potential of the microbial communities present in iron-rich hot springs, we conducted shotgun metagenomic ana­lyses. The bulk ana­lysis of metagenomic data showed that all sites possessed the potential for full carbon, nitrogen, and partial sulfur cycles (Fig. 5, 6A, and 6B). In Kowakubi hot spring, however, the distinct community structure brings unique functions related to carbon cycling, particularly in methane-related pathways. The pathway abundance table can be found in Table S3.

**Fig. 4.**
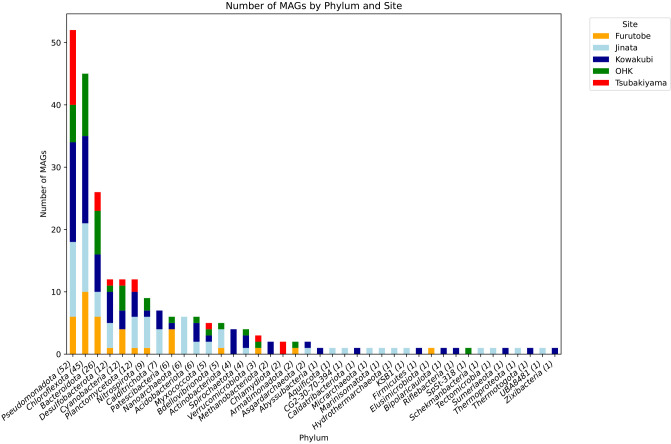
Number of metagenome-assembled genomes (MAGs) classified by phylum and site. The x-axis represents the identified phyla, with the number of MAGs for each phylum indicated in parentheses. The y-axis shows the number of MAGs per phylum. Sites are color-coded as follows: Furutobe (orange), Jinata (light blue), Kowakubi (dark blue), OHK (green), and Tsubakiyama (red). *Pseudomonadota* represents the most abundant phylum across all sites, followed by *Chloroflexota* and *Bacteroidota*.

A total of 242 medium- to high-quality MAGs (completeness ≥60%, contamination ≤5%) were reconstructed, identified, and annotated. They belonged to several phyla (Fig. 4). A list of the MAGs can be found in Table S4 and a summary of the functional annotations in Table S5. A tree with functional annotations can be found in Fig. S2. Of the MAGs, 185 are previously uncharacterized taxa spanning 87 MAGs identified only to family level, 25 MAGs identified only to order level and 9 MAGs identified only to class level, highlighting the novel diversity in the springs. We retrieved 12 Archaea MAGs only from Jinata and Kowakubi hot springs.

### Carbon cycling

**Fig. 5.**
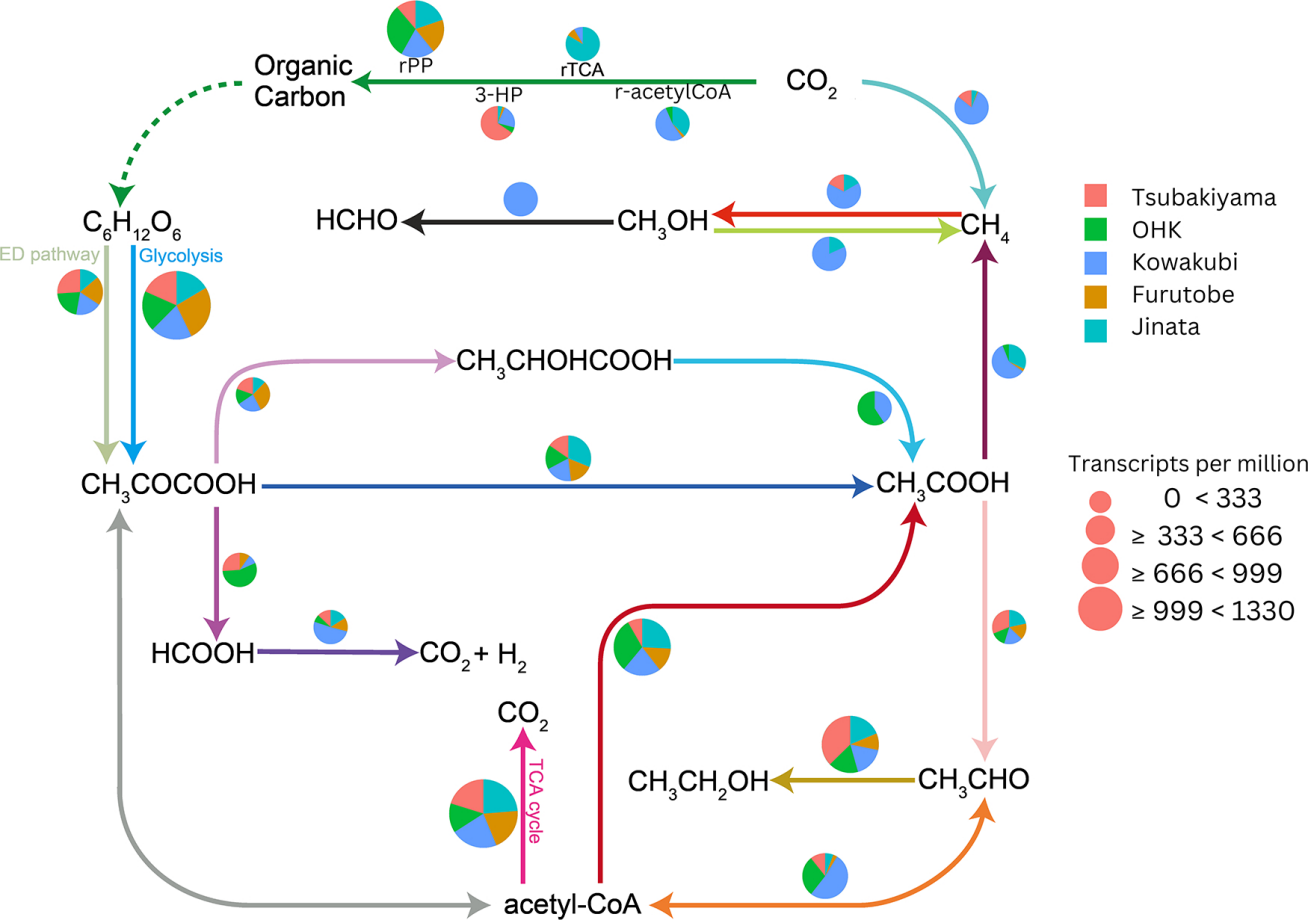
Overview of metabolic pathways in the carbon cycle across the iron-rich hot springs, generated with DiTing (Xue *et al.*, 2021). The diagram illustrates key metabolic intermediates and pathways, including the Entner-Doudoroff (ED) pathway, the reductive pentose phosphate pathway (rPP), the reductive tricarboxylic acid cycle (rTCA), the reductive acetyl-CoA pathway (r-acetylCoA), and the 3-hydroxypropionate bicycle (3-HP). Arrows indicate the direction of metabolic flux, with different colors representing distinct pathways. Pie charts represent the relative gene abundance at the hot springs: Furutobe (orange), Jinata (light blue), Kowakubi (dark blue), OHK (green), and Tsubakiyama (red). The size of the pie charts corresponds to gene abundances, normalized to transcripts per kilobase million (TPM).

Genes encoding for four of the eight known carbon fixation pathways (Garritano *et al.*, 2022) were detected from the genomic data of the springs: the reductive pentose phosphate cycle (rPP), the reductive tricarboxylic acid cycle (rTCA), the reductive acetyl-CoA pathway (r-acetylCoA), and the 3-hydroxypropionate bicycle (3-HP) (Fig. 5). Pathway abundance was calculated with the DiTing pipeline (Xue *et al.*, 2021) by normalizing the gene abundances to Transcripts Per Kilobase Million (TPM) and the relative abundance of each KO (KEGG Orthologs) necessary for each pathway (Xue *et al.*, 2021).

rPP is the dominant carbon‍ ‍fixation pathway across the sites and is utilized by‍ ‍bacteria of the *Cyanobacteria*, *Zetaproteobacteria*, *Hydrogenophilaceae*, and *Gallionellaceae* clades, which are the most abundant taxonomic groups present. The reductive acetyl-CoA pathway was the overall second most abundant carbon fixation pathway in the springs; it was most abundant at Jinata and Kowakubi, but was not present in Tsubakiyama hot spring. We observed methyl-coenzyme M reductase (*mcrABG*) genes involved in archaeal methane/alkane metabolism and other methane-related pathways, such as methyl-compound methyltransferases and methanol dehydrogenases, only at Kowakubi hot spring. Here, metabolic processes other than oxygenic photosynthesis and microaerophilic iron oxidation seem to be driving the microbial nutrient cycling. We performed a CCA ana­lysis comparing the geochemical parameters and abundance of genes related to specific metabolic functions involved in the biogeochemical cycling of C, N, and S (Fig. S1H). The CCA ana­lysis showed that DIC, DOC, and temperature were the main drivers of read abundance distribution, explaining ~41% of the variance. Genes encoding for both oxygenic and anoxygenic phototrophy show a negative association with DIC, DOC, and temperature, suggesting that higher levels hinder the relative abundance or activity of phototrophs in the springs. Genes encoding for methanogenesis, present in high abundances exclusively in Kowakubi, were positively associated with higher temperatures and DIC. Methanogens are apparently very thermo-adaptable for a single physiological group (Prondzinsky *et al.*, 2023), perhaps this adaptability results in an enrichment in comparison to other taxonomic groups. The rTCA pathway was observed in Kowakubi, Furutobe, and Jinata hot springs. Although we found genes related to the 3-HP bicycle present in all sites, it was more abundant in Tsubakiyama hot spring (Fig. 5).

### Carbon fixation and cycling as evidenced by MAG functional ana­lysis

Whereas the above discussion was derived from bulk metagenomic data, metagenome assembled genomes provide information on which taxonomic groups may be linked to these processes (Table S5, Fig. S2). A total of 57 MAGs encoded RuBisCo (Fig. 7) but carbon fixation via rPP was considered present only when the RuBisCo large subunit, phosphoglycerate kinase, glyceraldehyde-3-phosphate dehydrogenase, phosphoribulokinase, and transketolase genes were present.

A total of 40 MAGs encoded all considered genes for carbon fixation via rPP with different life modes, including aerobes, facultative anaerobes, and microaerophiles, with the capability to utilize ferrous iron, thiosulfate, sulfide, and/or hydrogen as electron donors. The recovered MAGs belonged to the phyla *Chloroflexota* (Jin0034, Jin00120, Jin00134, and Fur0044), *Pseudomonadota* (ɑ) (Kow0013, Kow0026, Kow0045, Kow00114, Kow00, and Tsb0032), *Pseudomonadota* (ɣ) (OHK0044, Tsb007, OHK0048, Jin0070, Tsb0026, Fur0039, Fur0019, Jin0032, Kow006, Kow0096, OHK0054, Jin00127, Fur0040, OHK0026, and Fur007), *Pseudomonadota* (ζ) (Jin004, Jin0012, Jin0030, and Jin0054), *Bacteroidota* (Jin002), *Calditrichota* (Jin0048), and *Cyanobacteria* (Fur006, Fur0025, Fur0042, Fur0046, OHK0012, OHK0035, OHK0037, OHK0047, Kow0091, and Tsb0014). Only the *Cyanobacteria* MAGs harbored photosynthesis genes.

All the MAGs involved in rPP carbon fixation had at least two out of three different types of terminal oxidases. These included: cytochrome c oxidase (*coxABCD*) present in facultative anaerobes (Pitcher and Watmough, 2004); cytochrome bd complex (*cydAB*) present in O_2_ limited conditions; and cytochrome c oxidase cbb3-type (*ccoPQNO*), prevalent in microaerophilic conditions (Sievert *et al.*, 2008; Jewell *et al.*, 2016). That is, the primary producers that utilize the rPP in the springs are adapted to the variable oxygen conditions. Moreover, the *Gammaproteobacteria* MAGs Kow006, Tsb0026, Kow0096, OHK0048, Jin0032, and Fur007 and the *Alphaproteobacteria* MAG Kow00114 encode the genes *norBC*, involved in nitric oxide reduction, and are, thus, also capable of anaerobic respiration, further increasing their adaptability.

Seven Archaeal MAGs encoded RuBisCo (Fig. S2), but none of them contained genes for phosphoribulokinase (*prk*) so it is unlikely that they are using rPP for carbon fixation. Although carbon fixation in Archaea with RuBisCo via an incomplete rPP cycle has been experimentally observed (Kono *et al.*, 2017), *prk* is still a requirement for it.

The reductive Acetyl-CoA pathway was considered complete when the enzymes acetyl-CoA decarbonylase/synthase complex subunit alpha or CO-methylating acetyl-CoA synthase, anaerobic carbon-monoxide dehydrogenase or aerobic carbon-monoxide dehydrogenase large subunit, formate dehydrogenase, formate-tetrahydrofolate ligase, methylenetetrahydrofolate dehydrogenase (NADP+)/methenyltetra­hydrofolate cyclohydrolase, and methylenetetrahydrofolate reductase (NADPH) were found in a MAG. Only one harbored all enzymes: Jin0080 from the phylum *Desulfobacterota*. However, we detected 12 MAGs which were missing only one enzyme, belonging to the phyla *Nitrospirota* (Kow0025), *Chloroflexota* (Jin00134, Kow0047, OHK0041), *Candidatus* Abyssubacteria (Kow0099), *Thermodesulfobacteriota* (Kow0054, Kow0092, Kow0089), *Actinomycetota* (Kow00129, Kow0056), *Myxococcota* (Jin00129), *SpSt-318* (OHK0040), and *Asgardarchaeota* (Kow0069). MAGs from the *Thermodesulfobacteriota* and *Nitrospirota* and one from *Actinomycetota* harbored oxygen-tolerant [NiFe]-hydrogenases Hyd-1 (Sargent, 2016). The observation of the reductive acetyl-CoA pathway in the *Actinomycetota* phylum and its validation is fairly recent and consistent with our findings (Merino *et al.*, 2020; Jiao *et al.*, 2021) the MAGs reconstructed here will aid in further understanding the evolution of it in this clade.

A total of 7 MAGs encoded complete rTCA cycle genes, from Kowakubi, Furutobe, and Jinata hot springs. They were phylogenetically diverse, from the *Bipolaricaulota* (Kow0097), *Myxococcota* (Kow0090), *Riflebacteria* (Kow0029; Notably, this MAG was missing only one gene required for a full rPP), *Nitrospirota* (Jin0064), *Aquificota* (Jin0029), *Elusimicrobiota* (Fur0012), and *Patescibacteria* (Fur0011). Several of these MAGs also harbored enzymes related to the nitrogen cycle, which will be discussed in the next section.

We found *mcrABG* genes involved in archaeal methane/alkane metabolism only in *Methanobacteriota* (Kow0021, Kow0019) MAGs from Kowakubi hot spring, a trend we saw for several other methane-related pathways, such as methyl-compound methyltransferases and methanol dehydrogenases (Table S5).

A recovered *Pseudomonadota* (β) (Fur0014) MAG from the genus *Rubrivivax* encodes sequences for the photosystem II reaction centre subunit H, but no carbon fixation genes were recovered. Other organisms closely related to *Rubrivivax* are known to perform photoheterotrophy (Nagashima *et al.*, 2012). Further studies to understand if this MAG is also capable of photoheterotrophy are necessary.

### Nitrogen cycling

**Fig. 6.**
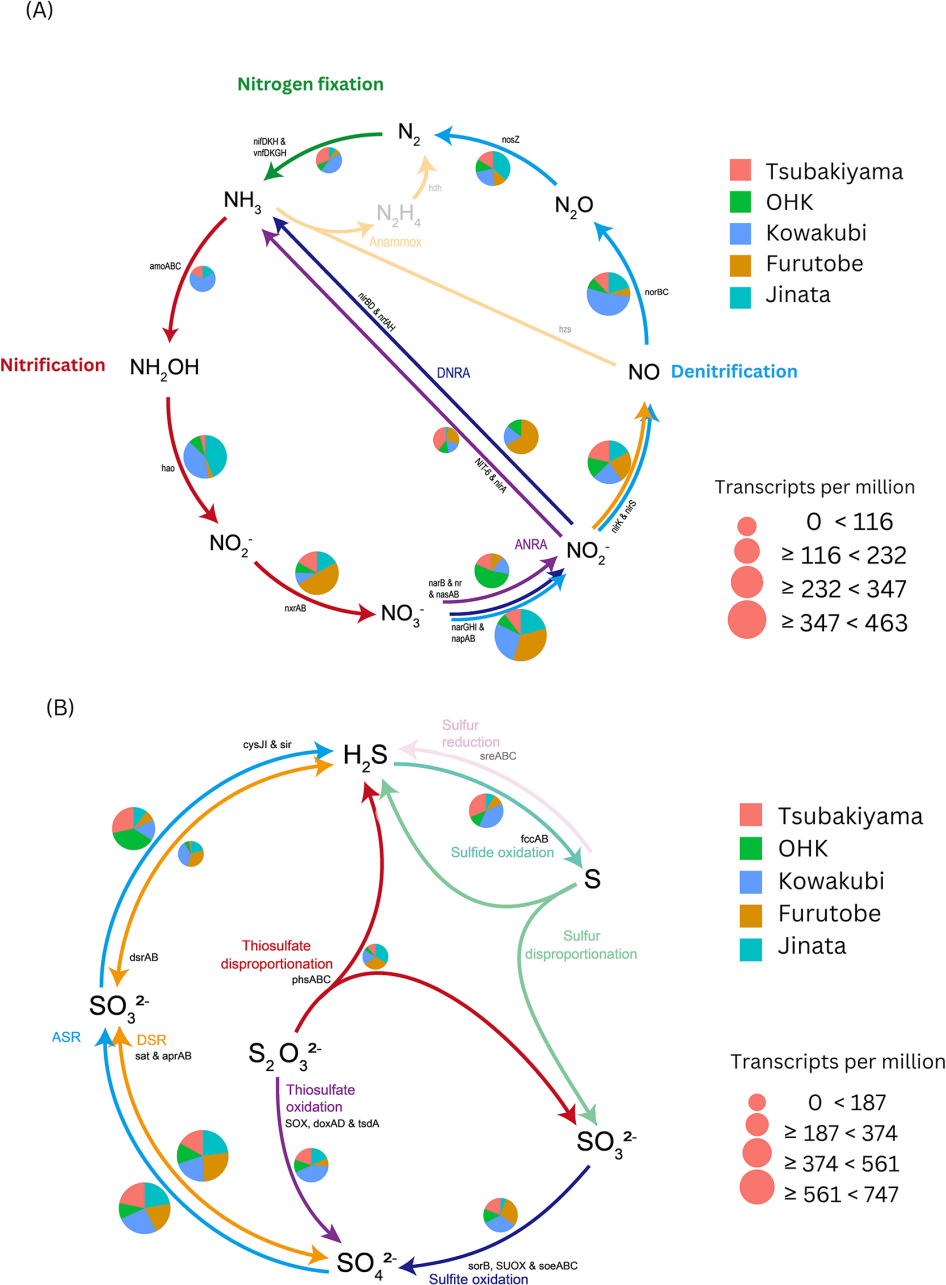
Overview of metabolic pathways in the nitrogen (A) and sulfur (B) cycles across the iron-rich hot springs, generated with DiTing (Xue *et al.*, 2021). (A) Nitrogen cycle: The pathways surveyed for nitrogen cycling include nitrogen fixation, nitrification, denitrification, dissimilatory nitrate reduction to ammonium (DNRA), assimilatory nitrate reduction to ammonium (ANRA), and anaerobic ammonium oxidation (anammox). Marker genes associated with each pathway are labeled alongside the arrows. Pie charts represent the relative gene abundances of these marker genes across the springs: Furutobe (orange), Jinata (light blue), Kowakubi (dark blue), OHK (green), and Tsubakiyama (red). The size of the pie charts corresponds to gene abundances normalized to transcripts per kilobase million (TPM). (B) Sulfur cycle: The sulfur cycling pathways include sulfide oxidation, sulfur disproportionation, thiosulfate oxidation and disproportionation, sulfur reduction, and sulfate reduction. The arrows indicate the direction of transformations between sulfur species, with marker genes for each pathway labeled. Pie charts, color-coded by site (as in panel A), illustrate the distribution of gene abundances for these marker genes.

We performed a bulk metagenome functional annotation and ana­lysis focused on genes involved in the N cycle, summarizing the results in Fig. 6A. We detected the capability for dissimilatory nitrate reduction to nitrite by nitrate reductase encoded in *narGHI* or *napAB* in all sites. The dissimilatory nitrate reduction to ammonia (DNRA) pathway (utilizing *nirBD* or *nrfAH* genes) was absent in Tsubakiyama and an order of magnitude lower in relative abundance in Jinata compared to the other springs. The genes for assimilatory pathways for nitrate reduction (*narB*,* NR*, *nasAB*, *nit-6*, and *nirA*) were present in all springs, but were again comparatively low at Jinata. The presence of nitrite reductase genes (*nirK* or *nirS*), nitric oxide reductase genes (*norBC*), and nitrous oxide reductase genes (*nosZ*) is ubiquitous and points to full denitrification in the springs. This suggests that the community as a whole might drive full denitrification, even if individual organisms only encode part of the genes necessary.

In aerobic ammonia oxidation, NH_3_-oxidizing bacteria (AOB) and archaea (AOA) oxidize NH_3_ via ammonia monooxygenase (AMO), encoded by *amoABC*, and produce nitrite via hydroxylamine oxidase (HAO), encoded by *hao* (Bellucci and Curtis, 2011; Nicol and Prosser, 2011). The nitrite produced is usually oxidized to nitrate by nitrite-oxidizing bacteria (NOB) via a nitrite oxidoreductase encoded by *nxrAB* (Daims *et al.*, 2016). The genes *amoABC* were found only in Tsubakiyama, Kowakubi, and Jinata, the high saline-temperature springs. This step is key in nitrification and is a process that necessitates oxygen (Wongchong and Loehr, 1975). The *hao* gene was found in all sites, but was more abundant in Jinata. Since the presence of *amoABC* genes is obligatory for aerobic ammonia oxidation, this brings forward the question of why *hao* genes were present across all the sites while *amo* genes were not. It is possible that aerobic ammonia oxidizers have low abundances, and therefore their functional genes are equally scarce and were not captured during amplification of the genetic material, or that annotation methods were insufficient even if several different annotation pipelines were utilized. Other possibilities are that there is an unknown mechanism, biochemical or abiotic, governing the oxidation of ammonia and generating hydroxylamine as a substrate for ammonia oxidizers, or that the *hao* gene product has a separate function. This is an area that requires further investigation.

In the absence of O_2_ to drive aerobic ammonia oxidation as the first step in nitrification, the anammox pathway has been reported in anaerobic environments. The anammox process involves the conversion of nitric oxide and ammonia to hydrazine and further to N_2_ utilizing enzymes transcribed by hydrazine synthase genes (*hzs*) and hydrazine dehydrogenase genes (*hdh*) (Kartal *et al.*, 2012). None of these genes were detected in the springs.

The transformation of nitrite to nitrate mediated by *nxrAB* was found in similar counts at all sites. Nitrogen fixation pathways using nitrogenase (*nifKDH* and/or *vfnDKGH*) were also present across all sites with varying abundances.

### Nitrogen fixation and cycling as evidenced by MAG functional ana­lysis

**Fig. 7.**
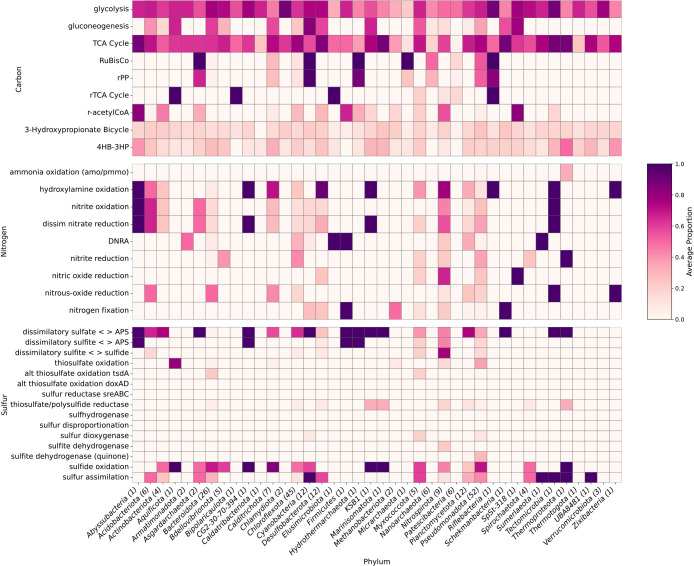
Overview of the metabolic functions annotated in the MAGs recovered from the iron-rich hot springs. The completeness of metabolic pathways involved in the carbon (C), nitrogen (N), and sulfur (S) cycles across microbial metagenome-assembled genomes (MAGs), grouped by phylum, is shown with a color gradient (0, no genes found—1, all necessary genes found). The rows represent specific metabolic pathways within the cycles. Microbial phyla are listed horizontally. A table with the functions found per MAG can be found in Table S5, for details on the genes selected for each pathway, see the KEGG-Decoder documentation (Graham *et al.*, 2018).

Nitrogen fixation was considered present when all three genes *nifHDK* were present in a MAG. No MAGs with *vfnDKGH* or *anfG* were found. We detected 12 MAGs encoding genes for nitrogen fixation (Fig. 7 and S2, Table S5) from the *Cyanobacteria* (Fur006, Kow00121, and OHK0047), *Desulfobacterota* (Jin0080, Jin0093, and Fur0034), *Firmicutes* (Kow00111), *Nitrospirota* (OHK006 and OHK0032), *Pseudomonadota* (ɑ) (Kow0026), *Pseudomonadota* (ɣ) (Kow0060), and *Schekmanbacteria* (Jin00124). Some MAGs also harbored at least one of the genes in the *nifHDK* operon and belonged to the *Planctomycetota* (Jin00125), *Myxococcota* (Jin00129), and *Cyanobacteria* (Tsb0014) (Fig. S2). The reconstructed MAGs harboring genes for diazotrophy belong to diverse clades and can use different electron donors and acceptors. They may contribute to steady nitrogen fixation under the variable conditions present in the springs by shifting their energy conservation mechanisms according to the environment.

There is no known *Planctomycetota* nitrogen-fixing representative, but their possible role in nitrogen fixation has been suggested (Kapili *et al.*, 2020). The *Planctomycetota* MAG reconstructed in this study (Jin00125) encodes *nifH* (though notably, this gene was not annotated by KBase-MicroTrait BioElement) and contributes to the growing body of evidence supporting this hypothesis.

Diazotrophy in *Firmicutes* was recently demonstrated in fermentative, thermophilic bacteria (Chen *et al.*, 2021). The *Firmicutes* MAG Kow00111, identified as *Thermincola ferriacetica*, is a chemolithoautotrophic ferric iron reducer (Zavarzina *et al.*, 2007) and has been studied for direct electrode reduction (Marshall and May, 2009), suggesting its potential role in both the iron and nitrogen cycles.

*amoABC* genes involved in aerobic ammonia oxidation were not found in the bulk metagenome data or MAGs of the low salinity springs OHK and Furutobe. We retrieved only two MAGs harboring *amoA/pmoA*: Kow0065 (*Pseudomonadota*) and Jin0077 (*Thermoproteota*). Strikingly, and in line with the bulk metagenome ana­lysis, 58 MAGs harbored the *hao* gene.

We reconstructed 9 MAGs from the *Nitrospirota* phylum (Fur0020, Jin00112, Jin0063, Jin0064, Jin0073, Jin0085, Kow0025, OHK0032, and OHK006), from which members of the genus *Nitrospira* are known for performing complete ammonia oxidation (commamox) utilizing *amoABC*, *hao*, and *nxrAB* genes (van Kessel *et al.*, 2015). In none of the reconstructed *Nitrospirota* MAGs *amoABC* was found. However, in Kow0025, Fur0020, and OHK0032, both *hao* and *nxrAB* were present. Their specific role in the nitrogen cycle remains enigmatic and further studies are needed to elucidate this matter.

Widespread potential for denitrification was present across the sites with 127 of the MAGs containing one or more key genes selected as markers of denitrification, including nitrate reductase (*narGHI*), nitrite reductase (*nirS/nirK*), nitric oxide reductase (*norBC*), and nitrous oxide reductase (*nosZ*). Some MAGs harbored all the enzymes necessary for complete denitrification, while others possessed partial pathways. The MAGs belonged to the *Pseudomonadota*, *Bacteroidota*, *Chloroflexota*, *Nitrospirota*, *Cyanobacteria*, *Desulfobacterota*, *Planctomycetota*, *Bdellovibrionota*, CG2-30-70-394, *Acidobacteriota*, KSB1, *Tectomicrobia*, *Calditrichota*, *Actinobacteriota*, *Zixibacteria*, *Abyssubacteria*, *Spirochaetota*, *Myxococcota*, and SpSt-318. This and the bulk metagenome ana­lysis results indicate that denitrification is a common metabolic capability among the microbial communities present in the studied iron rich hot springs.

### Sulfur cycling

Dissimilatory and assimilatory sulfate reduction transforms sulfate to sulfide. Sulfate is converted to adenosine 5′-phosphosulfate (APS) via sulfate adenylyltransferase and sulfate reductase, encoded by *sat* and *cysJI* respectively, which were found in all springs (Fig. 6B). APS is reduced to sulfite via APS reductase encoded by *aprAB* (Speich *et al.*, 1994), and subsequently sulfite is reduced to sulfide via sulfite reductase encoded by the *sir* gene. Both were present in all springs. In the assimilatory pathway, the sulfide can then be utilized for the biosynthesis of cysteine and methionine (Kushkevych *et al.*, 2020). Genes for the last step in dissimilatory sulfate reduction (*dsrAB*), which produces sulfide, were also found in all springs at variable abundances (Fig. 6B). The absence of genes for sulfur reduction to sulfide (*sreABC*), sulfur disproportionation (*SOR*), and sulfhydrogenase suggests that the organisms at these sites may not be able to utilize elemental sulfur. Thiosulfate conversion genes mediated by the SOX complex, *doxAD*, *phsAB*, and *tsdA* were present in all sites, and point to the importance of‍ ‍thiosulfate as an electron donor in iron-rich hot springs. Thiosulfate can be used as an electron donor coupled with the reduction of oxygen (aerobic conditions) (Harrold *et al.*, 2016; Twible *et al.*, 2024) or nitrate/Fe^3+^ (anaerobic conditions) (Jørgensen, 1990; Teske *et al.*, 2000; Ding *et al.*, 2023) as well as by some phototrophic organisms (Hensen *et al.*, 2006; Marshall and Morris, 2013).

The relative abundances of sulfotrophic genes/microbes in the springs suggest an active sulfur cycle despite sulfate concentrations being moderate to low and sulfide not being detected. How then is S being cycled in these springs and what insights can we gain from them about early Earth?

A similar phenomenon of sequence ana­lysis suggesting the presence of microbial sulfate reduction, but lack of chemical traces of the metabolism has been described in the Chilean oxygen minimum zone (OMZ), where a cryptic sulfur cycle is present (Canfield *et al.*, 2010). High rates of sulfate reduction in the OMZ are followed by fast microbial-sulfide-oxidation coupled with nitrate reduction to nitrite, effectively removing sulfide from solution. In the springs studied here, titration with ferrous iron could be removing reduced sulfur species, including sulfur and sulfide, produced by the organisms (Sanden *et al.*, 2021). Because in these hot springs *Cyanobacteria* and iron-oxidizing bacteria are present, both biotic and abiotic oxidation of iron occurs. The iron oxyhydroxides could later interact with sulfide minerals and release again ferrous iron and thiosulfate (and other intermediate sulfur species) into the environment (Vera *et al.*, 2022). Indeed, thiosulfate oxidation and disproportionation genes were found in all springs irregardless of the sulfate concentrations. In the context of early Earth, evidence of microbial mats and weathering has been observed in ancient (~3.4 billion-year-old) detrital sedimentary pyrite grains (Wacey *et al.*, 2011). Bulk geochemical measurements are not sufficient to unfold the interactions occurring in the springs between microbes, sulfur, and ferrous iron. Further experimental validation using sulfur isotope tracers will be vital to our understanding of the interaction between Fe^2+^-rich waters, microbial metabolism, and S cycling on early Earth, as well as the evidence of it that might (or not) be found in ancient rocks.

### Sulfur cycling as evidenced by MAG functional ana­lysis

We reconstructed 176 MAGs that contained one or more key genes involved in sulfur assimilation (*sir* and *cysJ*), dissimilatory sulfate reduction (*sat*, *aprAB*, and *dsrAB*), sulfite dehydrogenase (*sorB* and *soeABC-quinone*), and/or sulfide oxidation (*sqr* and/or *fccB*) (Fig. 7 and S2, Table S5). Other pathways identified included thiosulfate oxidation (*soxABCDEFG*) and thiosulfate dehydrogenase (*tsdA*). Sulfur reductase (*sreABC*) genes involved in sulfur reduction and sulfur oxygenase/reductase (*SOR*) genes, involved in aerobic sulfur disproportionation (Wang *et al.*, 2023), were absent in all MAGs.

Twelve MAGs from the *Pseudomonadota* encoded for *soxBCY*, (ɑ) (Tsb0032, Kow00114, Kow0032, Tsb0019, Kow0045, Kow0013, and Tsb0024), and (ɣ) (Tsb007, OHK0048, Fur007, Kow0065, and Jin00127) and all of those also harbored *sqr/fccB*. Two of these MAGs, unidentified to species level, belonged to the *Rhodoferax* genus (Fur007 and OHK0048). Other *Rhodoferax* species have been found to oxidize and reduce iron, use H_2_, Fe^2+^, and thiosulfate as electron donors with O_2_ as the electron acceptor, and perform carbon fixation via rPP (Kato and Ohkuma, 2021). Only Fur007 was found to code genes for iron oxidation and Fe reduction genes were not observed in either of the two *Rodoferax* MAGs. However, genes for microaerobic respiration, RuBisCo, and nitrate reduction were observed, suggesting that these MAGs also have a role in the S, C, N, and potentially Fe cycle.

Some MAGs possessed complete pathways, while others exhibited partial or intermediate steps in sulfur compounds reduction and oxidation. Notably, several *Pseudomonadota* MAGs from the inland hot spring Kowakubi and the ocean-side hot spring Tsubakiyama (Kow00114, Tsb0032, Kow0045, Tsb0024, and Kow0058) showed the presence of dimethylsulfoniopropionate (DMSP) demethylation genes (*dmdA*), indicating the potential for sulfur utilization in various forms. Yet, no DMSP biosynthesis genes were recovered.

Six *Cyanobacteria* MAGs (Tsb0014, Kow0091, Kow0049, Fur006, and OHK0047) contained Type A heme-copper oxygen reductase genes (*coxAB*) for aerobic respiration and cytochrome bd oxidase genes (*cydAB*) for microaerobic respiration. They also encoded the sulfide quinone oxidoreductase (*sqr*) gene, which has been reported in all *Cyanobacteria* capable of anoxygenic photosynthesis with sulfide as the reductant. SQR is also utilized for sulfide detoxification (Arieli *et al.*, 1994; Hamilton *et al.*, 2018). This suggests that the *Cyanobacteria* present in the iron-rich hot springs studied here are well adapted to changes in oxygen concentration and might be involved in anoxygenic photosynthesis in the presence of sulfide or, at the very least, have detoxification mechanisms for sulfide that also enhance their adaptability to the springs changing conditions.

### Iron cycling

**Fig. 8.**
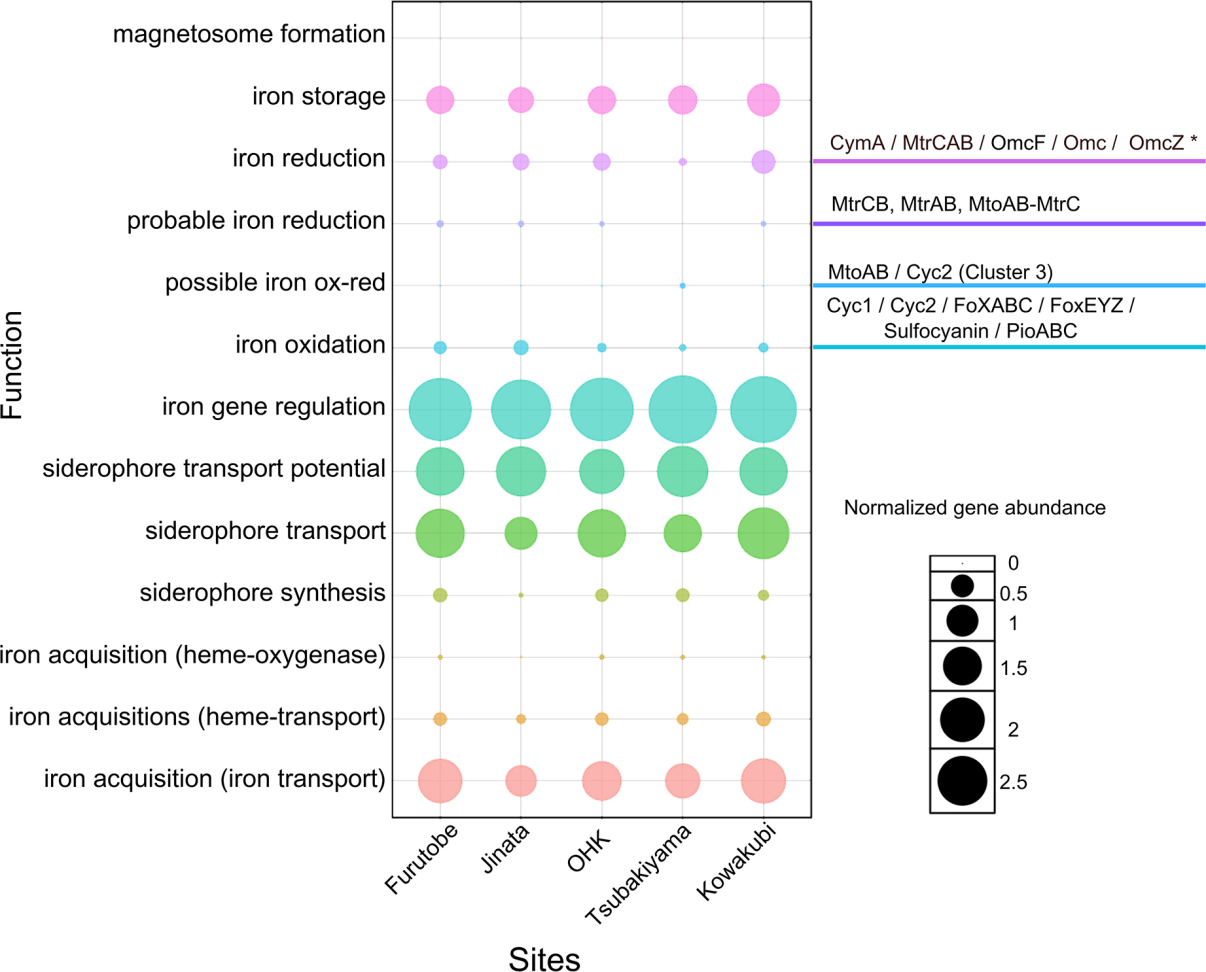
Bubble plot showing the normalized gene abundance associated with iron metabolism across iron-rich hot springs: Furutobe, Jinata, OHK, Tsubakiyama, and Kowakubi. The surveyed genes for iron reduction and oxidation are on the right side. For a table of the numerical values see Table S6, and for details on the software/hmm profiles see the documentation of FeGenie (Garber *et al.*, 2020). For a heatmap of each gene in the sites see Fig. S3. The size of each bubble corresponds to the normalized abundance of genes from each iron category identified in each site, normalized to the number of predicted open reading frames (ORFs). This normalized value is then multiplied by an inflation factor of 1,000 for easier visualization to generate the plotted values.

Genes related to iron oxidation and reduction were found in all sites, suggesting the presence of a full iron cycle mediated by microbes (Fig. 8). Regulation of iron uptake is necessary for microorganisms (Andrews *et al.*, 2003), and an ana­lysis on the iron cycling genes in the springs revealed that iron-sensory genes and those involved in transcriptional-level regulation of iron uptake, such as *fur*, *dtxR*, and *fecR*, were the most abundant (Fig. S3). The next most abundant category was siderophores, related to iron acquisition, transport, and storage, further highlighting the importance of iron homeostasis in the cells. A heatmap of the abundances of each gene per site can be found in Fig. S3. The siderophore synthesis gene families *vabB*, *vabB*, *vabC*, and *vabE* (Balado *et al.*, 2006) were detected only at OHK, while *rhbD* and *rhbE* (Lynch *et al.*, 2001) were found only at Jinata and Kowakubi (Fig. S3).

Genes related to iron oxidation and reduction followed distribution patterns similar to those of the iron-utilizing microbial groups, suggesting that although the functions are equivalent, the specific enzymes utilized for each process might differ according to the geochemical conditions/microbial community structure. The genes *mtoA* and *mtrB*, involved in electron transfer during iron respiration (Coursolle and Gralnick, 2010), were found solely on Tsubakiyama hot spring.

The *cyc1* genes involved in electron transfer were only detected in Furutobe and a BLAST ana­lysis showed the sequences were related to Burkholderiales cytochromes, however they were not identified at a species level. Cyc2 is a cytochrome utilized by neutrophilic iron oxidizers, both saline and non-saline (McAllister *et al.*, 2020). Genes for the *cyc2* cluster No. 2 were found only at Tsubakiyama. A BLAST search showed the sequences belonged to *Zetaproteobacteria* members (and one to *Nitrosospira multiformis*), suggesting the *Zetaproteobacteria* diversity found here is distinct from that of Jinata, where *Zetaproteobacteria* are also abundant according to our amplicon sequencing ana­lysis. The *cyc2* gene clusters No. 1 and No. 3 were observed at Jinata, OHK, and Furutobe. Only cluster No. 3 of *cyc2* was found at Kowakubi. FoxE, part of the *foxEYZ* operon, confers bacteria the capability to perform light-dependent Fe (II) oxidation, and was detected at all sites at very low abundances. Bacteriochlo­rophylls and reaction center-encoding genes related to anoxygenic photosynthesis, such as *pufM*, were also scarcely found. Although anoxygenic phototrophs that utilize sulfide or ferrous iron as electron donors are often invoked as primary producers on early Earth (Camacho *et al.*, 2017), in the Precambrian analogues studied here we found a scarcity of functional genes for anoxygenic photosynthesis reaction centers.

**Fig. 9.**
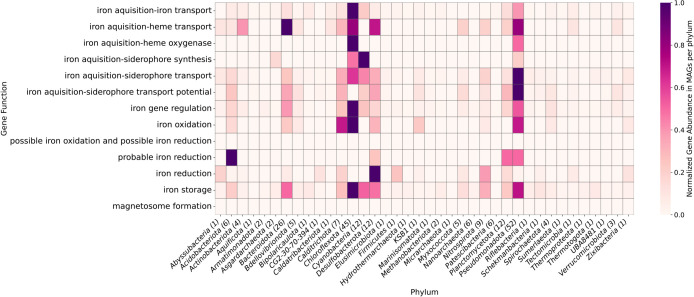
Heatmap showing the normalized abundance of iron-related genes across metagenome-assembled genomes (MAGs), grouped by phylum. The y-axis lists key iron-related metabolic functions. The x-axis represents individual MAGs, grouped by their corresponding phylum. The color intensity represents the normalized gene abundance. The normalized gene abundance was calculated as the number of genes identified for each function in a MAG, divided by the total predicted open reading frames (ORFs) for that MAG, and then scaled by an inflation factor of 1000. These values were rescaled to a 0 to 1 range within each gene category to facilitate comparisons while maintaining the relative abundance within each category. A table of the values in each MAG can be found in Table S7.

All recovered MAGs had at least one gene related to iron acquisition, transport, or utilization as identified by FeGenie (Fig. 9 and S2, Table S5). Similar to the bulk metagenomic annotations, the iron genes found in these MAGs are most abundantly related to regulation and siderophore synthesis. Among the MAGs identified as involved in iron oxidation (Table 2), thirteen belong to the *Anaerolineae* class from the phylum *Chloroflexota*. While iron oxidation is not widely recognized in *Anaerolineae*, their presence in this group suggests a potential yet understudied role in iron cycling. With this ana­lysis, we were also able to identify the iron oxidizing bacteria that are capable of autotrophy (Fig. S2), which encompass not just the Classes *Zetaproteobacteria* and *Gammaproteobacteria*, but also the *Calditrichota*. In total, 41 MAGs encoded for iron oxidation genes (Table 2) and 33 MAGs harbored iron reduction genes (Table 3).

## Conclusions

What type of microbial communities were present on the early Earth as the atmosphere became oxygenated, yet the oceans remained iron-rich? One hypothesis that prevails regarding the primary producers on early Earth is that during the Archean, methanogens and anoxygenic phototrophs were the main players, giving way to oxygenic phototrophs after the advent of oxygenic photosynthesis (Ozaki *et al.*, 2019; Watanabe *et al.*, 2023). The controls of early primary productivity are controversial, but considered to be nutrient limitation (Alcott *et al.*, 2022; Crockford and Halevy, 2022), electron donor scarcity (Ward *et al.*, 2019b), niche-limitation (*e.g.*, iron toxicity in *Cyanobacteria*, but the paradoxical necessity of it in photosystems) (Shcolnick *et al.*, 2009; Enzingmüller‐Bleyl *et al.*, 2022), and geological processes, such as continental crust growth (Olejarz *et al.*, 2021; Stevenson, 2023). We suggest that the proliferation of oxygenic photosynthesis expanded the available electron acceptors for microaerophilic chemolithotrophs, who were able to exploit the initially low oxygen concentrations and reduced compound availability, enhancing primary productivity and acting as a sink for oxygen. Except for Kowakubi hot spring, in which apparently non-iron related metabolic processes are dominant, the iron-rich hot springs surveyed contain high abundances of iron oxidizing bacteria (FeOB) capable of carbon fixation using the reductive pentose phosphate cycle, who together with *Cyanobacteria* were the‍ ‍most abundant primary producers. The FeOB groups were associated with different taxonomic lineages: *Zetaproteobacteria* and/or *Caldithrichia* at Jinata, Tsubakiyama, and Kowakubi and *Gammaproteobacteria* at Furutobe and OHK. Notably, members of the *Anaerolineae* capable of iron oxidation were ubiquitous. Despite these taxonomic differences, they perform equivalent ecological functions within their respective environments.

In most sites, the FeOB had higher relative abundances than *Cyanobacteria*. This supports the idea that under the analyzed conditions, microaerophilic iron oxidizers can not only outcompete abiotic Fe oxidation, but they can co-exist with *Cyanobacteria* and use the microoxic conditions perhaps to their advantage (*e.g.*, reduced substrate competition between CO_2_ and O_2_ at RuBisCo). Oxygenic phototrophic *Cyanobacteria* are not outcompeting chemolithotrophs, but‍ ‍could be seen as fueling communities dominated by microaerophilic iron oxidizers from the *Zetaproteobacteria*, *Gammaproteobacteria*, *Chloroflexota*, and *Calditrichota* clades, among others. The rapid utilization of oxygen by these organisms might also promote growth of species sensitive to oxygen by removing reactive oxygen species from the environment. Although genes related to sulfide oxidation/detoxification and sulfate assimilation were found in the metagenomes, the hot springs are neither sulphidic nor sulfate rich, which suggests the potential for a cryptic sulfur cycle in which medium-to-low sulfate levels promote the growth of organisms capable of recycling several forms of sulfur compounds. The observation of *Cyanobacteria* in high temperature/iron environments in this and other studies also expands our understanding of the distribution of the clade and suggests that a higher tolerance for iron could mean a broader distribution in the iron-rich early Earth’s oceans. However, even in springs where *Cyanobacteria* were not abundant, rather than anoxygenic phototrophs, chemolithotrophs were the most abundant microbial group. All the springs in this study have the potential to support complete carbon, nitrogen, and iron cycles as well as a partial sulfur cycle. According to bulk metagenome annotations, these cycles occur in roughly equal proportions, though they are carried out by different taxonomic groups in each spring.

These iron-rich hot springs provide a unique natural laboratory to study microbial metabolism under early Earth-like conditions, expanding our understanding of the dynamics between oxygenic and chemolithotrophic primary productivity. Unlike previous studies that mainly focused on *Cyanobacteria* or anoxygenic phototrophs, our approach revealed that FeOB from the *Zetaproteobacteria*, *Gammaproteobacteria*, *Chloroflexota*, and *Calditrichota*, have vital roles in primary production under microoxic conditions, questioning prevailing assumptions about early primary productivity. The co-occurrence of FeOB and *Cyanobacteria* suggests that microbial iron oxidation influences oxygen availability and carbon fixation strategies. Additionally, the observed functional redundancy across taxonomic groups points to some stability in the biogeochemical cycles of iron-rich hot spring systems. By characterizing these microbial interactions under their geochemical context, this study not only enhances our understanding of modern iron-rich ecosystems, but also provides a framework for interpreting biosignatures in the rock record, guiding future research on early Earth. The iron-rich hot springs studied here can be used to understand the mechanisms driving biogeochemical cycles on early Earth, and call for further studies that focus on analyzing the biosignatures of the metabolic pathways active, microbial activity rates, and putative cryptic cycles to further guide our interpretations of the rock record.

### Accession numbers

The sequenced data was deposited in the DNA Data Bank of Japan (DDBJ) under the accession number PRJDB18100. The supplementary tables and full-sized figures are available on FigShare (https://figshare.com/projects/Metabolic_Potential_and_Microbial_Diversity_of_Late_Archean_to_Early_Proterozoic_Ocean_Analog_Hot_Springs_of_Japan/235073).

## Citation

Li-Hau, F., Nakagawa, M., Kakegawa, T., Ward, LM., Ueno, Y., and McGlynn, S. E. (2025) Metabolic Potential and Microbial Diversity of Late Archean to Early Proterozoic Ocean Analog Hot Springs of Japan. *Microbes Environ ***40**: ME24067.

https://doi.org/10.1264/jsme2.ME24067

## Supplementary Material

Supplementary Material

## Figures and Tables

**Table 1.** Geochemical conditions of five iron rich hot springs of Japan and the modern ocean.

Site	Location	Temperature (°C)	DO (μM)	pH	Fe(II) (μM)	Conductivity (mS)	TDS (g L^–1^)	Salinity (ppt)	DIC (mM)	DOC (mg L^–1^)	δ13C-DIC (vs VPDB)	δ13C-DOC (vs VPDB)	SO_4_^2–^ (mM)
**Jinata**	34°19′04.4″N 139°12′57.3″E	55.3*	75.9*	5.77*	41.9*	37.5	41	22.3	15	22.6	2.01	–26.04	17*
**Tsubakiyama**	40°35′39.72″N, 139°51′55.39″E	51.7	25	7.5	88.6	39.9	45	24.2	9.7	7.2	1.14	–22.84	24.81 (Takashima *et al.*, 2010)
**OHK**	40°24′28.66″N, 140°45′16.96″E	44	50	6.5	71.9	4.6	3.51	2.4	11.2	4.3	2.09	–16.28	5.5 –7.9 (Takashima *et al.*, 2010; Shiraishi *et al.*, 2022)
**Furutobe**	40°25′49.9″N 140°40′13.4″E	44	15.6	6.3	87.2	4.9	3.75	2.6	15.4	5	2.17	NA	7–8.3 (Takashima *et al.*, 2010; Shiraishi *et al.*, 2022)
**Kowakubi**	39°33′30.9″N 140°16′57.4″E	50	16.2	7	74.5	33.5	42.6	20.2	12.2	9.4	5.82	–22.32	<1 (Testing Center Kozakura, 2018)
**Modern Ocean water (mean values)**	—	3.5 (Boyle, 2007)	250 (Song *et al.*, 2019)	8.1 (Cole, 2013)	0.0005 (Lauderdale *et al.*, 2020)	50 (Boyle, 2007)	35 (Boyle, 2007)	35 (Boyle, 2007)	2.4 (Cole, 2013)	0.6 (Kim *et al.*, 2015)	0.89 (Cheng *et al.*, 2019)	–22 (Kim *et al.*, 2015)	28 (Fakhraee *et al.*, 2024)

* Averaged values at the source from Ward *et al.* (2019a) and tidal variations recorded in this study. Other Jinata values are averaged from measurements at the source at different tides. ppt: parts per thousand.

**Table 2.** MAGs recovered from iron-rich hot springs that have genes utilized in iron oxidation^1^

Phylum	MAGs
*Calditrichota*	Jin0095, Jin0048, Kow00108, Kow0037, Kow0042
*Pseudomonadota*	Fur0039, Fur007, Jin0012, OHK0026, Jin0030, Jin004, Kow0096
*KSB1*	Jin0045
*Desulfobacterota*	Jin00111, Kow00128, Fur0034
*Chloroflexota*	Fur0018, Jin00109, OHK0010, Jin00120, Fur0016, Fur0017, Fur002, Jin0031, OHK003, Jin0068, Kow0070, Kow0077, Kow0080, Jin0081
*Bacteroidota*	Jin00101, Fur0015, Kow0098
*Spirochaetota*	Kow00102
*Myxococcota*	Tsb0020
*Nitrospirota*	OHK0032, Jin0064
*Bdellovibrionota*	Jin0041
*Acidobacteriota*	Jin0044, Jin0089
*Zixibacteria*	Kow0074

^1^ The genes encoding for the protein families Cyc1, Cyc2, FoXABC, FoxEYZ, Sulfocyanin, and PioABC were used for detecting iron oxidation

**Table 3.** MAGs recovered from iron-rich hot springs that have genes utilized in iron reduction^1^

Phylum	MAGs
*Nitrospirota*	Kow0025, OHK006, Jin00112, Jin0073
*Firmicutes*	Kow00111
*Desulfobacterota*	OHK0028, Jin009, Fur0034, Kow0092, Kow00128, Tsb0017, Kow0054, Kow0089, Jin00111, Kow00100, Jin0080, Jin0093
*Abyssubacteria*	Kow0099
*SpSt-318*	OHK0040
*Calditrichota*	Jin0095, Kow0037
*Zixibacteria*	Kow0074
*Planctomycetota*	Jin0010, Fur0037
*CG2-30-70-394*	Jin0039
*Myxococcota*	Jin0088
*Bacteroidota*	Kow0098
*Tectomicrobia*	Jin0047
*KSB1*	Jin0045
*Sheckmanbacteria*	Jin00124
*Pseudomonadota*	Jin004
*Actinobacteriota*	Kow0056
*Acidobacteriota*	Kow0062

^1^ The genes encoding for the protein families CymA, MtrCAB, OmcF, OmcS, OmcZ, FmnA-dmkA-fmnB-ppIA-ndh2-eetAB-dmkB, DFE_0448-0451, DFE_0461-0465, MtrCB, MtrAB, and MtoAB-MtrC were used for detecting iron reduction.
