# Supplementary material for: Metabolic Potential and Microbial Diversity of Late Archean to Early Proterozoic Ocean Analog Hot Springs of Japan
Source: Microbes Environ. 2025 Jul 23;40(3):ME24067. doi: 10.1264/jsme2.ME24067 (PMC12501874; doi:10.1264/jsme2.ME24067)
Supplement: Supplementary file 1 — Supplementary Material [file 40_24067_s1.pdf]

(Li-Hau et al., 2025)

Metabolic potential and microbial diversity of late Archean to early Proterozoic ocean analog hot springs of Japan

Figure S1A

Relative abundance plot for Bacteria at Phylum level based on 16S rRNA amplicon sequencing per sample, with each site in a separate panel

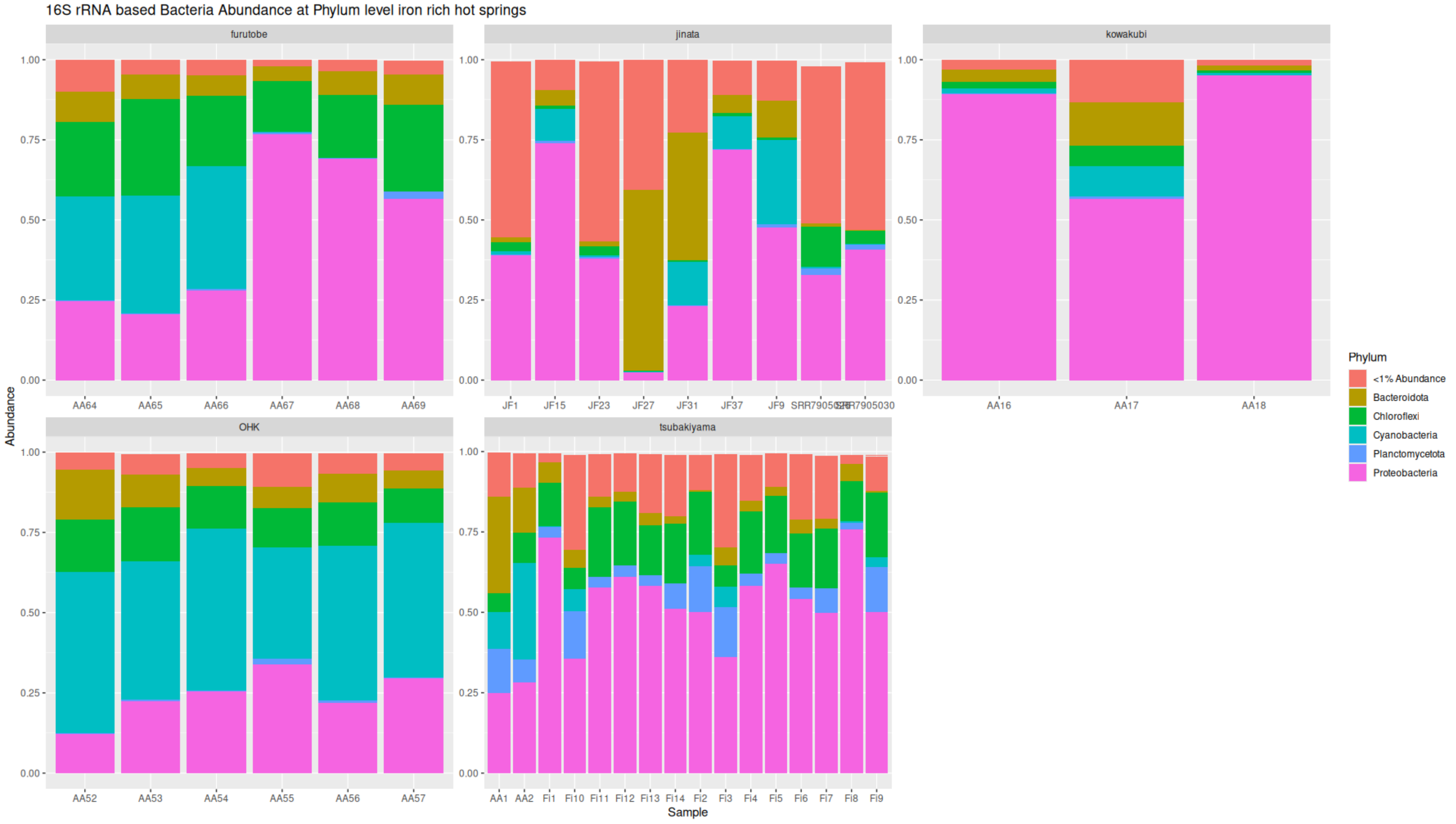

Figure S1B 16S Relative abundance plot at Phylum level for Archaea based on rRNA amplicon sequencing per sample and per site

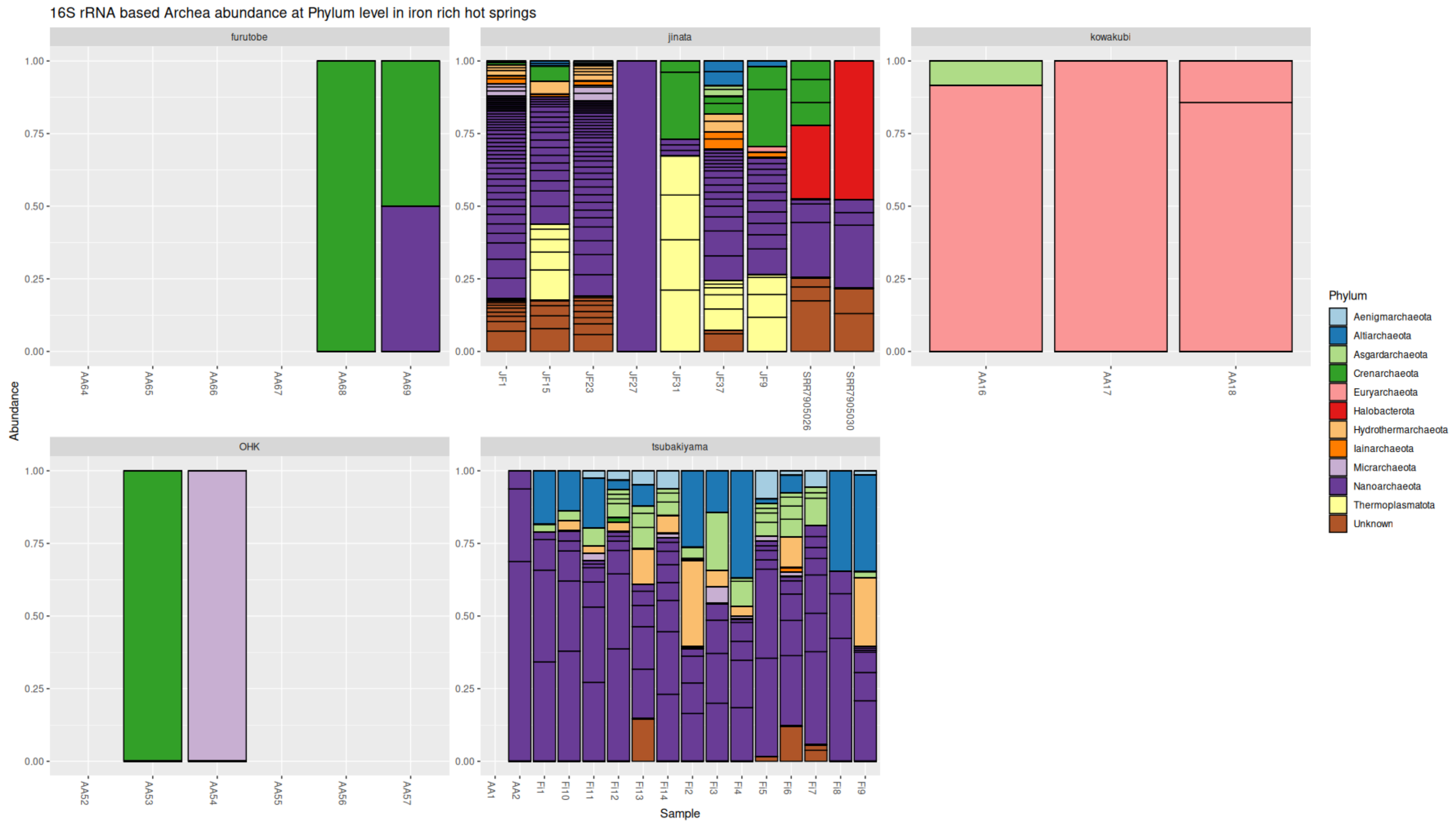

Figure S1C
Principal Coordinate Analysis of Bray-Curtis distances on 16S rRNA amplicon samples from iron rich hot springs of Japan (Rarefaction samples)

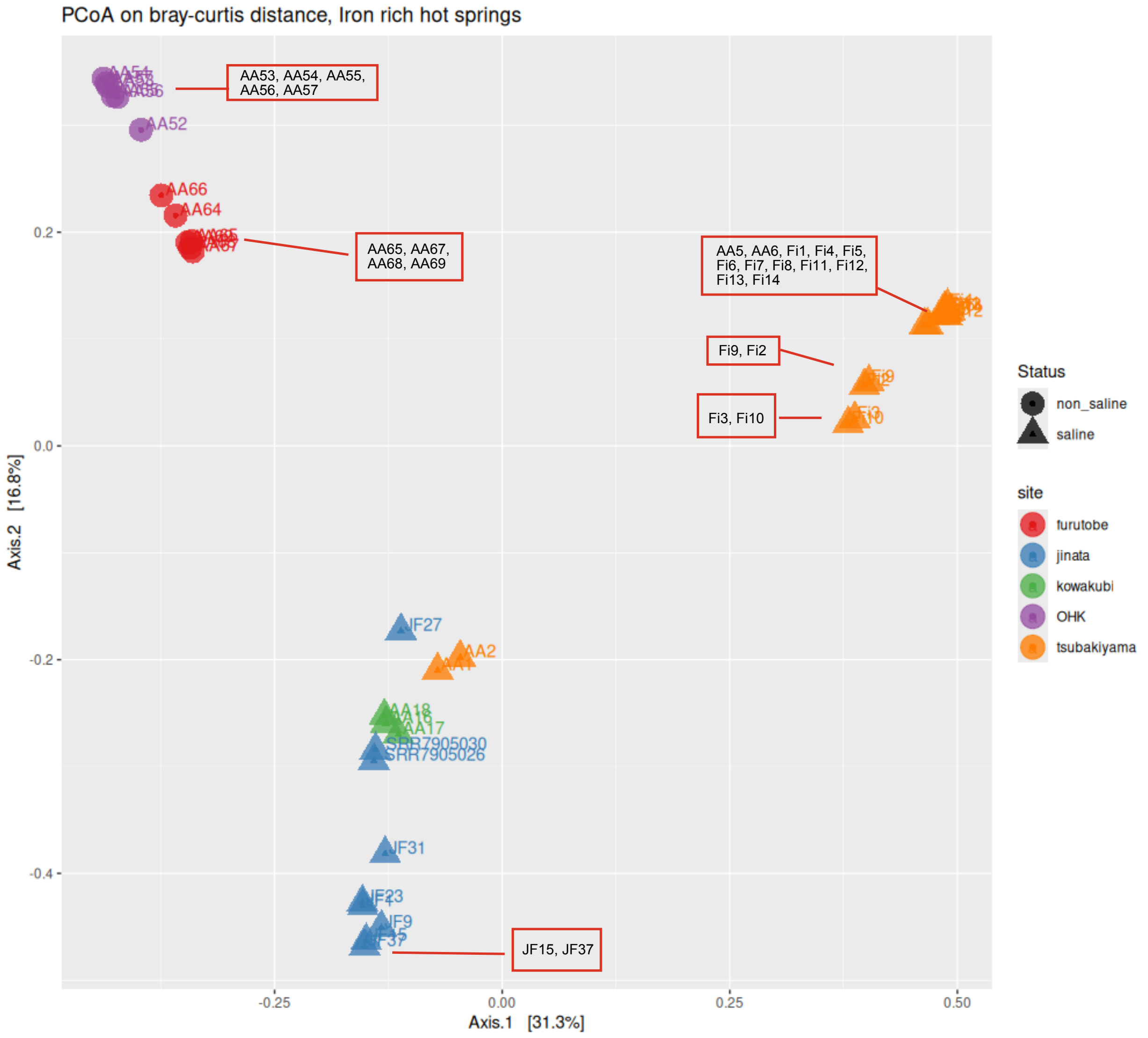

Principal Coordinates Analysis (PCoA) plot based on Bray-Curtis distances, illustrating the compositional variation of microbial communities across iron-rich hot spring sites. The analysis was performed on microbial community data from five sites: Furutobe (red circles), Jinata (blue triangles), Kowakubi (green diamonds), OHK (purple squares), and Tsubakiyama (orange triangles). Symbols represent samples, with their shapes indicating environmental salinity status: triangles for saline environments and circles for non-saline environments.Axis 1 explains 31.3% of the variance, while Axis 2 accounts for 16.8%, capturing a significant portion of the community structure variation. Clear clustering patterns highlight site-specific community compositions, with saline samples (triangles) generally clustering separately from non-saline samples (circles) within their respective sites. The figure underscores the influence of salinity and site-specific conditions on microbial community structure in these unique environments.

Figure S1D

Principal Coordinate Analysis of unweighted unifrac distances of 16S rRNA amplicon samples from iron rich hot springs of Japan (Rarefaction samples)

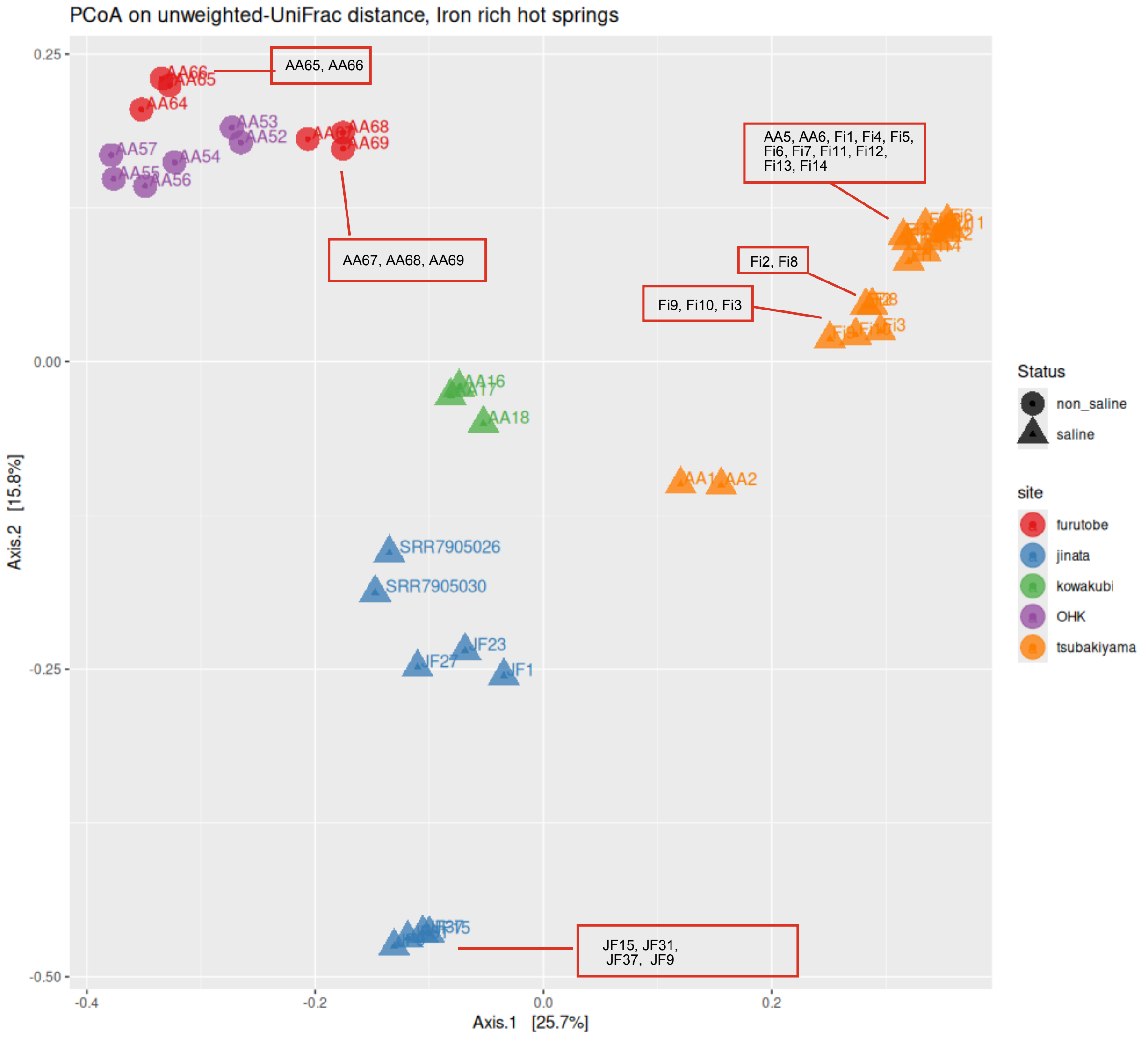

Principal Coordinates Analysis (PCoA) plot based on unweighted UniFrac distances, illustrating the beta diversity of microbial communities across iron-rich hot spring sites. The analysis compares microbial community composition among samples collected from five distinct sites: Furutobe (red circles), Jinata (blue triangles), Kowakubi (green triangles), OHK (purple circles), and Tsubakiyama (orange triangles). Circles denote non-saline environments, and triangles denote saline environments.The two axes explain the majority of the observed variation in the dataset, with Axis 1 accounting for 25.7% of the variation and Axis 2 explaining 15.8%. The clustering patterns observed indicate site-specific microbial community compositions, with clear segregation between communities from saline and non-saline environments. This result highlights the significant influence of both site location and salinity on microbial community structure in these iron-rich hot springs.

Figure S1E
Principal Coordinate Analysis of weighted unifrac distances of 16S rRNA amplicon samples from iron rich hot springs of Japan (Rarefaction samples)

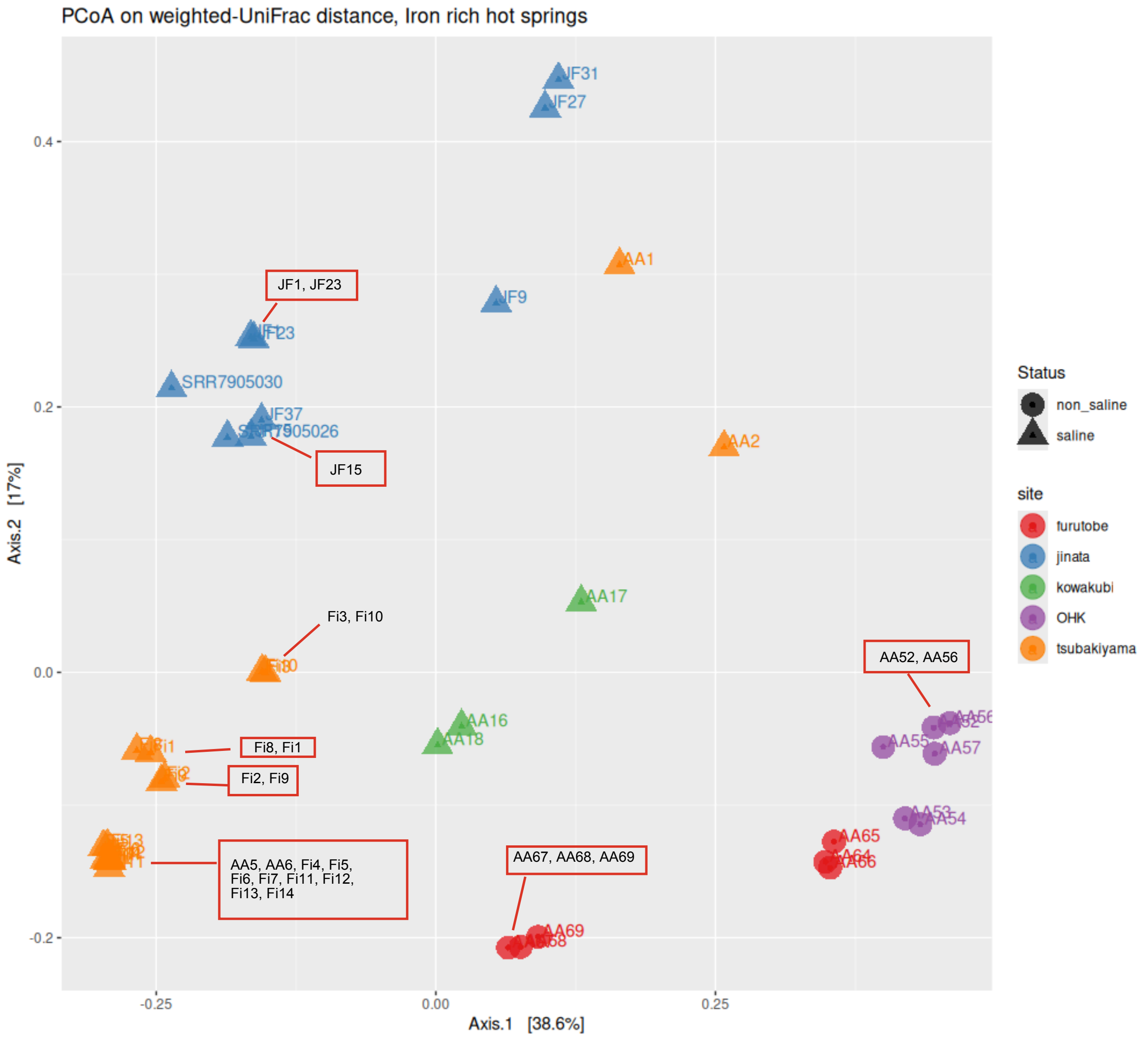

Principal Coordinates Analysis (PCoA) plot based on weighted UniFrac distances, illustrating the beta diversity of microbial communities across iron-rich hot spring sites. Weighted UniFrac distances, account for differences in the abundance of shared and unshared lineages, providing insight into how community composition varies in terms of dominant taxa.The plot compares microbial community composition among samples from five distinct sites: Furutobe (red circles), Jinata (blue triangles), Kowakubi (green triangles), OHK (purple circles), and Tsubakiyama (orange triangles). Circles indicate non-saline environments, and triangles indicate saline environments.The two axes together explain a substantial portion of the observed variation in microbial community composition, with Axis 1 accounting for 38.6% of the variation and Axis 2 explaining 17.0%. Samples are clustered based on site-specific and salinity-driven differences, demonstrating the distinct microbial community structures present in each hot spring environment. The weighted UniFrac distances emphasize the influence of dominant taxa and relative abundances, highlighting both site-specific and environmental effects on microbial community composition.

Figure S1F
Hierarchical clustering analysis of geochemical parameter of iron rich hot springs of Japan.
Parameters included: Temperature (ºC), DO (µM), pH, Fe(II) (µM), Conductivity (mS), TDS (g/L), Salinity (ppt), DIC (mM), DOC (mg/L), and δ13C -DIC (vs VPDB)).

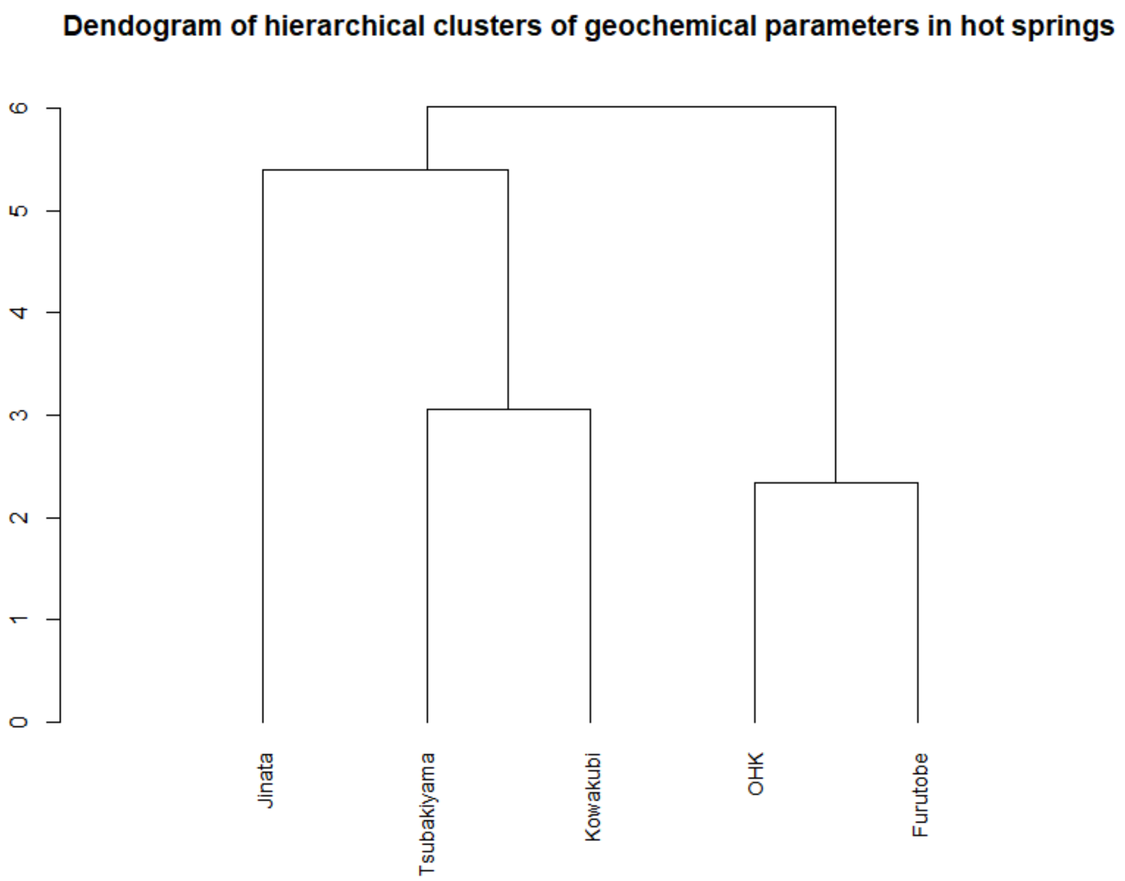

Figure S1G

Alpha diversity plots of unrarefied 16S rRNA amplicon sequencing samples from iron rich hot springs of Japan.

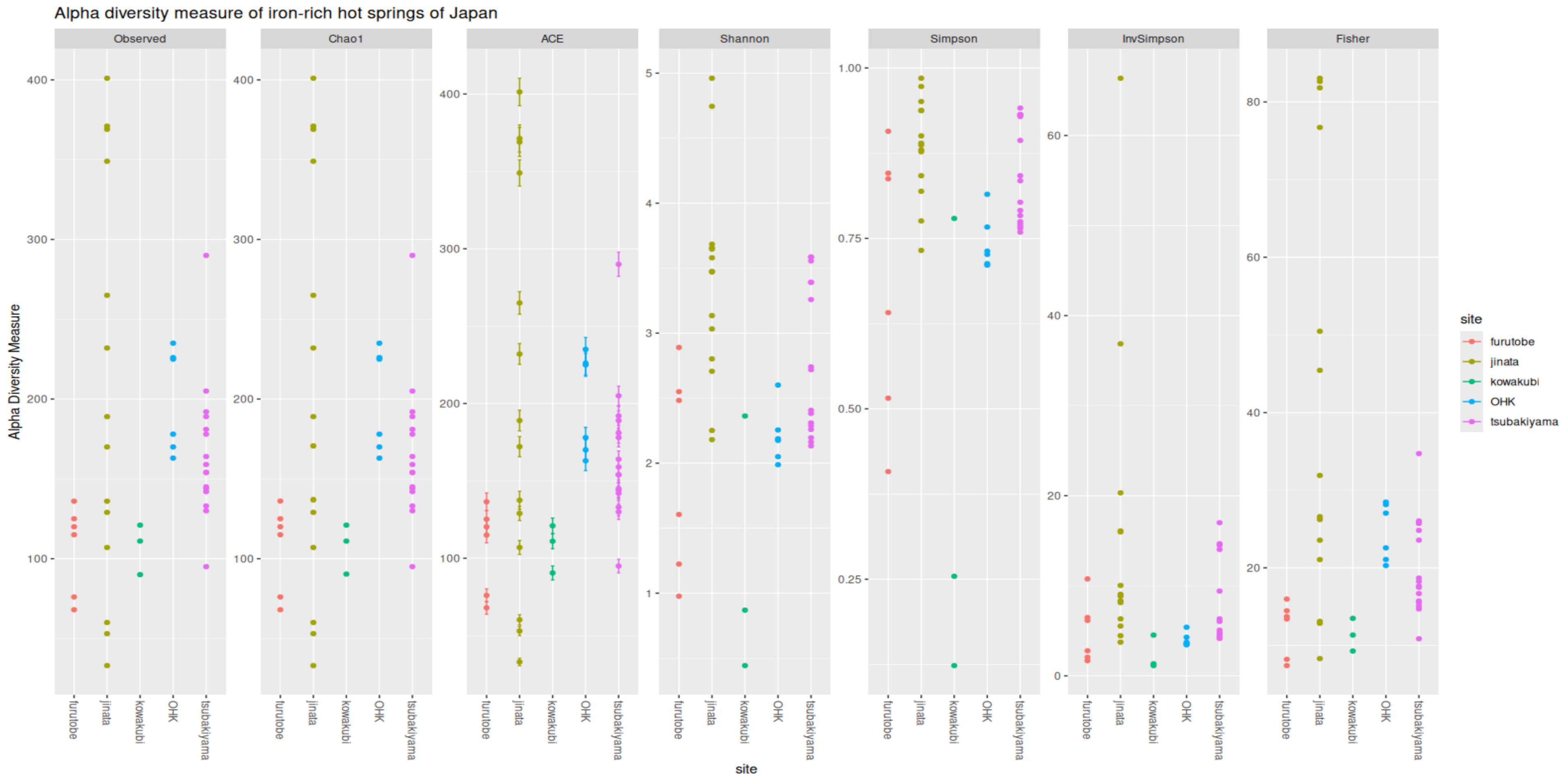

The Simpson index (Simpson 1949) suggests high dominance in Kowakubi. In contrast, Jinata and Tsubakiyama have higher index values, suggesting that species are well-distributed. Although the most abundant species found at OHK and Furutobe are similar, the richness at OHK is higher in all tested metrics, particularly in Chao1 index, suggesting OHK has more rare taxa in low numbers. It is necessary to point out that recent studies have suggested that the Chao1 index should not be used when amplicon sequence data is denoised with DADA2, as the algorithm removes singletons that are vital in calculations of this index (Deng et al. 2024).

Figure S1H CCA analysis on metabolic functions and geochemical parameters samples from iron rich hot springs of Japan based on read abundances of genes in the bulk metagenome

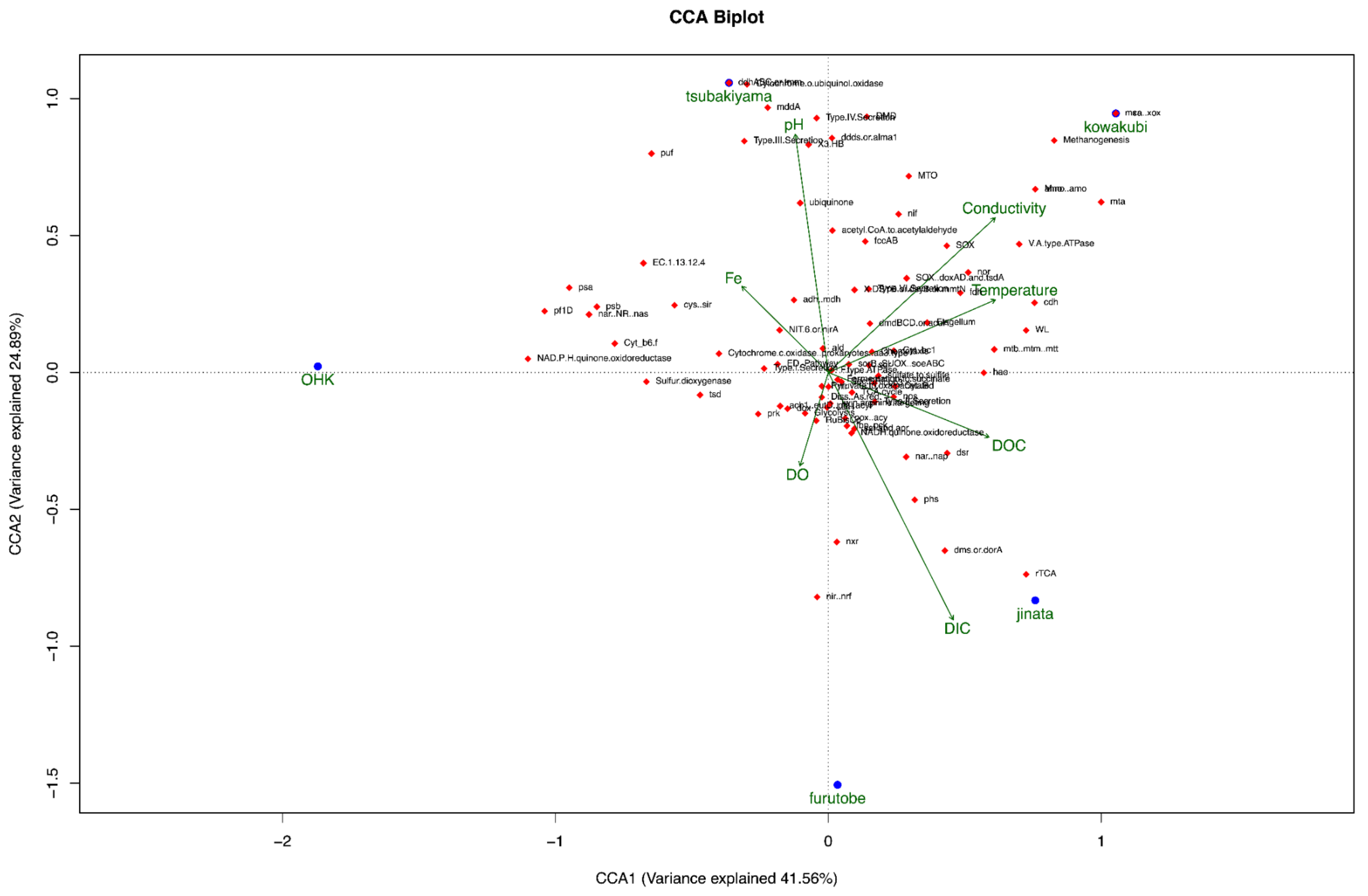

The biplot depicts microbial functional genes (red diamonds) and site (blue and green labels) in relation to geochemical parameters (green vectors). The primary axes, CCA1 and CCA2, explain 41.56% and 24.89% of the variance, respectively. Vectors represent geochemical variables, with their direction and length indicating the strength and direction of their influence on microbial community composition and function.Key geochemical parameters, including pH, conductivity, temperature, dissolved oxygen (DO), dissolved inorganic carbon (DIC), and dissolved organic carbon (DOC), show varied impacts across the sites.

Figure S1I
PCA analysis on 16S rRNA amplicon sequences and geochemical parameters on iron rich hot springs of Japan.

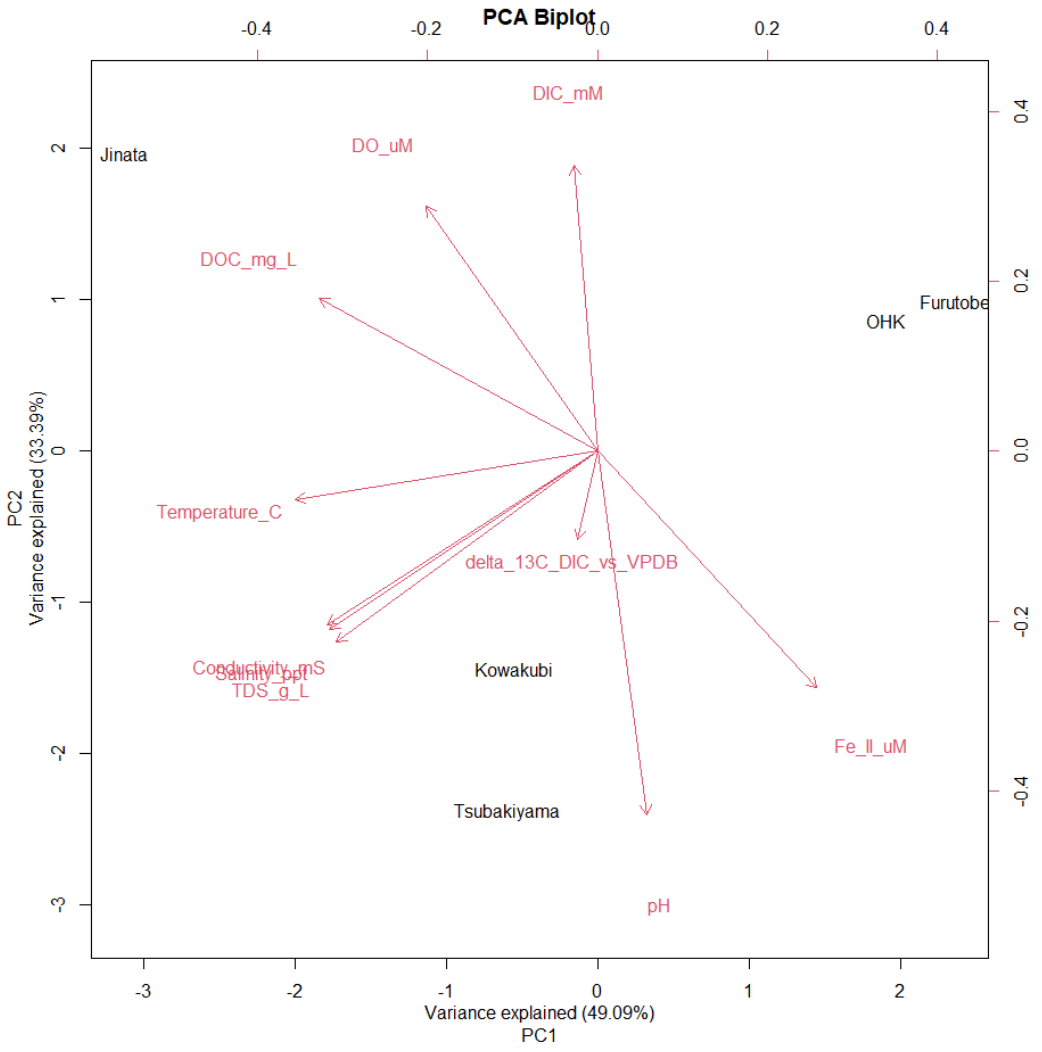

Principal Component Analysis (PCA) biplot of environmental variables from iron-rich hot spring sites, illustrating relationships among sites and key environmental factors. The analysis was performed to identify the major gradients driving variation in environmental conditions across five hot spring sites: Furutobe, Jinata, Kowakubi, OHK, and Tsubakiyama.The first principal component (PC1) explains 49.09% of the variance, while the second principal component (PC2) explains 33.39%, capturing a combined total of 82.48% of the variability in the dataset. The arrows represent environmental variables, with their direction and length indicating the strength and influence of each variable on the principal components. Environmental parameters include pH, temperature (°C), dissolved organic carbon (DOC, mg/L), dissolved inorganic carbon (DIC, mM), iron (Fe(II), µM), salinity (ppt), total dissolved solids (TDS, g/L), and $\delta^{13}C$ of DIC. Jinata aligns with elevated temperature and DOC levels, while Furutobe and OHK are influenced by higher DIC and Fe(II) concentrations. Tsubakiyama correlates with higher pH, while Kowakubi is associated with higher salinity and TDS.

Figure S1J

Loadings of PCA analysis on 16S rRNA amplicon sequences and geochemical parameters on iron rich hot springs of Japan.

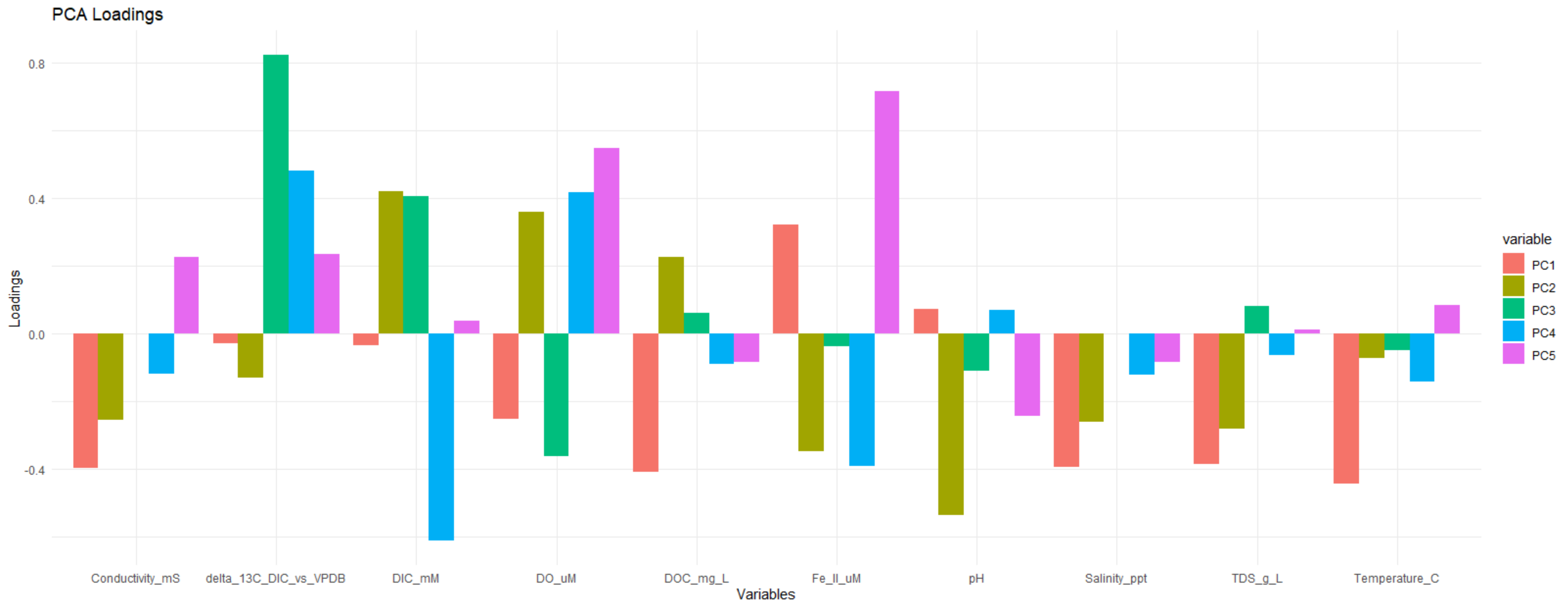

Loadings of Principal Component Analysis (PCA) performed on 16S rRNA amplicon sequences and geochemical parameters from iron-rich hot springs in Japan. This bar plot represents the contribution of each environmental variable to the first five principal components (PC1 to PC5). The loadings indicate how strongly each variable correlates with a given principal component, highlighting its importance in explaining variability across the dataset. The x-axis lists the geochemical variables, including conductivity (mS), $\delta^{13}C$ of DIC (δ13C_DIC_vs_VPDB), dissolved inorganic carbon (DIC, mM), dissolved oxygen (DO, µM), dissolved organic carbon (DOC, mg/L), iron (Fe(II), µM), pH, salinity (ppt), total dissolved solids (TDS, g/L), and temperature (°C). The y-axis shows the loading values, where positive and negative values indicate the direction of correlation with each principal component. PC1 (red) and PC2 (orange) explain the largest variance in the data, with PC1 heavily influenced by DIC and DOC concentrations, while PC2 shows strong correlations with temperature and salinity. Higher principal components (PC3 to PC5) explain smaller variances, with distinct contributions from variables such as pH and $\delta^{13}C$ of DIC. This analysis provides insight into the multivariate relationships between microbial community compositions and environmental parameters across the studied hot springs

Figure S2. Unrooted Phylogenetic tree of Metagenome Assembled Genomes reconstructed from iron-rich hot springs of Japan

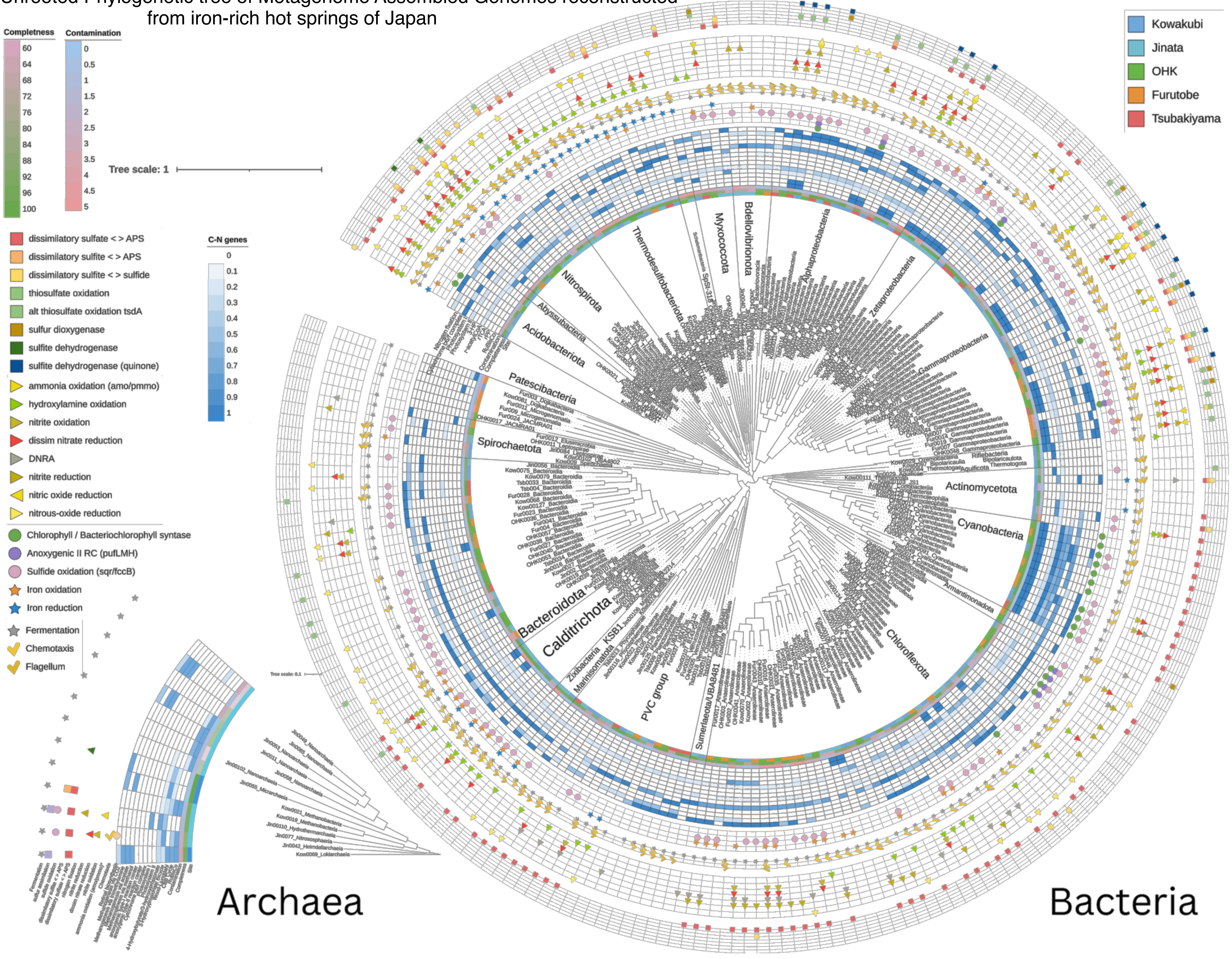

Supplementary Figure S2. Unrooted Phylogenetic tree of Metagenome Assembled Genomes reconstructed from iron-rich hot springs of Japan. The tree was generated using the Genome Taxonomy Database Toolkit (GTDB-Tk) (Chaumeil, P. A, et al., 2019) from an alignment of 120 bacterial and 122 archaeal marker genes. MAGs are represented as leaf nodes, with taxonomic classifications at the phylum level as text labels.

Outer rings show the hot spring name, MAG completeness, contamination, and functional gene annotations. Completeness and contamination were estimated using CheckM (Parks et al., 2015). Functional gene annotations are from KEGG-Decoder (Graham et al., 2018) and FeGenie (Garber et al., 2020). Values for nitrogen and carbon fixation, are shown in a heatmap ring. Gradients in shades of blue indicate the completeness of the metabolic pathways. Figure annotations (squares, triangles, stars, checkmarks) represent presence or absence for other functional categories. Pathways are marked as present if at least 50% of the genes required for a complete pathway were detected. *amoa/pmo completeness is 33%, but was added as only one subunit was detected. For a high quality figure please go to:
(https://figshare.com/projects/
Metabolic_Potential_and_Microbial_Diversity_of_Late_Archean_to_Early_Proterozoic_Ocean_Analog_Hot_Springs_of_Japan/
235073).

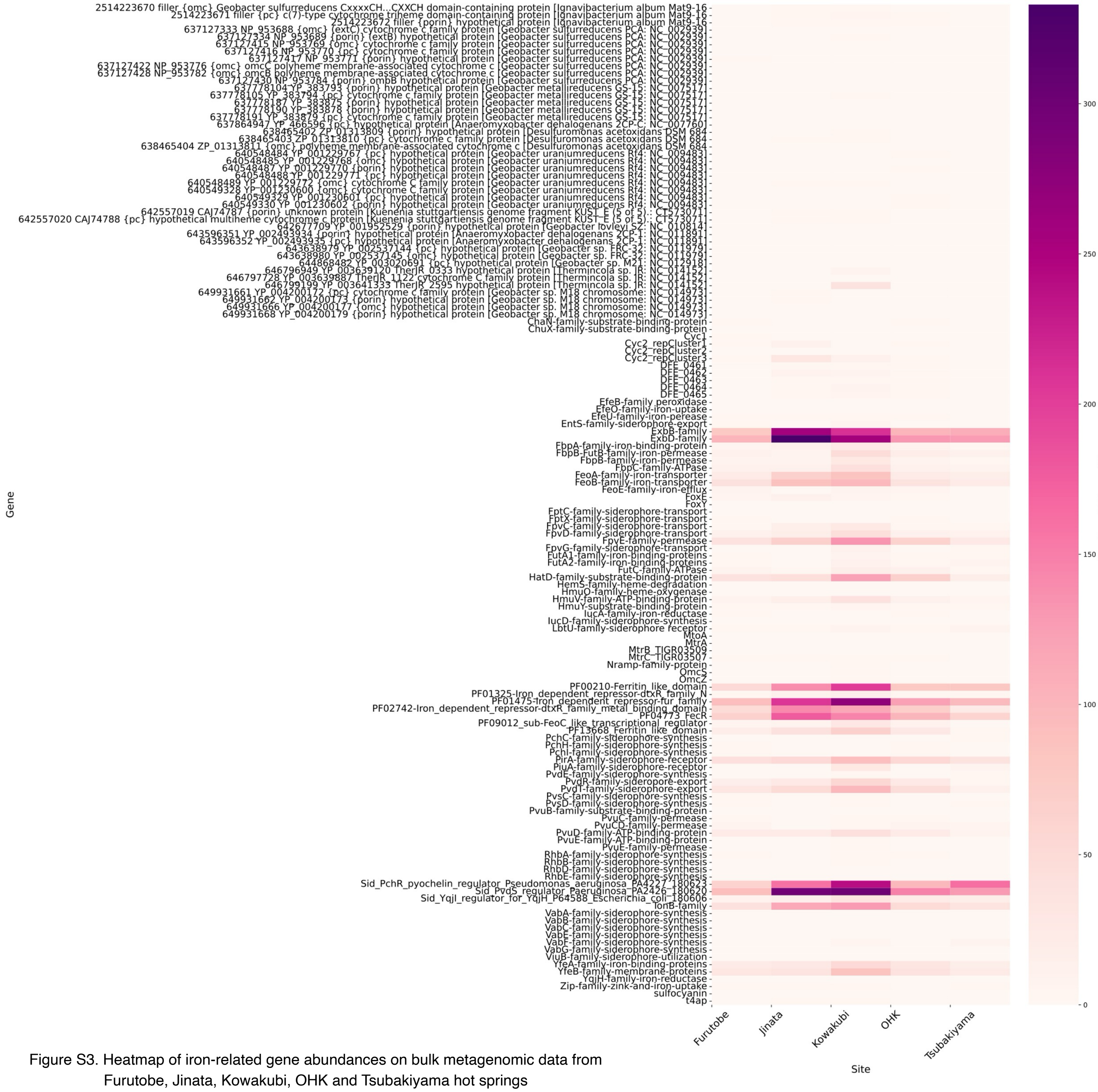

Figure S3. Heatmap of iron-related gene abundances on bulk metagenomic data from Furutobe, Jinata, Kowakubi, OHK and Tsubakiyama hot springs
